# Cognitive, functional, and neuropsychiatric correlates of regional tau pathology in autopsy-confirmed chronic traumatic encephalopathy

**DOI:** 10.1186/s13024-023-00697-2

**Published:** 2024-02-06

**Authors:** Michael L. Alosco, Micaela White, Carter Bell, Farwa Faheem, Yorghos Tripodis, Eukyung Yhang, Zachary Baucom, Brett Martin, Joseph Palmisano, Kristen Dams-O’Connor, John F. Crary, Lee E. Goldstein, Douglas I. Katz, Brigid Dwyer, Daniel H. Daneshvar, Christopher Nowinski, Robert C. Cantu, Neil W. Kowall, Robert A. Stern, Victor E. Alvarez, Bertrand Russell Huber, Thor D. Stein, Ann C. McKee, Jesse Mez

**Affiliations:** 1 Boston University Alzheimer’s Disease Center and CTE Center, Boston University Chobanian & Avedisian School of Medicine, Boston, MA USA; 2 Department of Neurology, Boston University Chobanian & Avedisian School of Medicine, Boston, MA USA; 3 Davis School of Medicine, University of California, Sacramento, CA USA; 4 Department of Neurology, New York University Grossman School of Medicine, New York, NY USA; 5 Department of Biostatistics, Boston University School of Public Health, Boston, MA USA; 6 Biostatistics and Epidemiology Data Analytics Center, Boston University School of Public Health, Boston, MA USA; 7 Department of Rehabilitation and Human Performance, Brain Injury Research Center, Icahn School of Medicine at Mount Sinai, New York, NY USA; 8 Department of Neurology, Icahn School of Medicine at Mount Sinai, New York, NY USA; 9 Department of Pathology, Molecular and Cell-Based Medicine, Icahn School of Medicine at Mount Sinai, New York, NY USA; 10 Department of Pathology and Laboratory Medicine, Boston University Chobanian & Avedisian School of Medicine, Boston, MA USA; 11 Department of Psychiatry, Boston University Chobanian & Avedisian School of Medicine, Boston, MA USA; 12 Departments of Biomedical, Electrical & Computer Engineering, Boston University College of Engineering, Boston, MA USA; 13 Braintree Rehabilitation Hospital, Braintree, MA USA; 14 Department of Physical Medicine and Rehabilitation, Harvard Medical School, Boston, MA USA; 15 Concussion Legacy Foundation, Boston, MA USA; 16 Department of Neurosurgery, Boston University Chobanian & Avedisian School of Medicine, Boston, MA USA; 17 Department of Neurosurgery, Emerson Hospital, Concord, MA USA; 18 Department of Anatomy and Neurobiology, Boston University Chobanian & Avedisian School of Medicine, Boston, MA USA; 19 System, U.S. Department of Veteran Affairs, VA Boston Healthcare, Boston, MA USA; 20 Department of Veterans Affairs Medical Center, Bedford, MA USA

**Keywords:** Activities of daily living, Amygdala, Behavioral dysregulation, Chronic traumatic encephalopathy, Clinicopathological correlation, Cognition, Frontal cortex, Tau, Temporal cortex, Traumatic brain injury

## Abstract

**Background:**

Chronic traumatic encephalopathy (CTE) is a neurodegenerative disease characterized by hyperphosphorylated tau (p-tau) accumulation. The clinical features associated with CTE pathology are unclear. In brain donors with autopsy-confirmed CTE, we investigated the association of CTE p-tau pathology density and location with cognitive, functional, and neuropsychiatric symptoms.

**Methods:**

In 364 brain donors with autopsy confirmed CTE, semi-quantitative p-tau severity (range: 0–3) was assessed in 10 cortical and subcortical regions. We summed ratings across regions to form a p-tau severity global composite (range: 0–30). Informants completed standardized scales of cognition (Cognitive Difficulties Scale, CDS; BRIEF-A Metacognition Index, MI), activities of daily living (Functional Activities Questionnaire), neurobehavioral dysregulation (BRIEF-A Behavioral Regulation Index, BRI; Barratt Impulsiveness Scale, BIS-11), aggression (Brown-Goodwin Aggression Scale), depression (Geriatric Depression Scale-15, GDS-15), and apathy (Apathy Evaluation Scale, AES). Ordinary least squares regression models examined associations between global and regional p-tau severity (separate models for each region) with each clinical scale, adjusting for age at death, racial identity, education level, and history of hypertension, obstructive sleep apnea, and substance use treatment. Ridge regression models that incorporated p-tau severity across all regions in the same model assessed which regions showed independent effects.

**Results:**

The sample was predominantly American football players (333; 91.2%); 140 (38.5%) had low CTE and 224 (61.5%) had high CTE. Global p-tau severity was associated with higher (i.e., worse) scores on the cognitive and functional scales: MI ($\beta$_standardized_ = 0.02, 95%CI = 0.01–0.04), CDS ($\beta$_standardized_ = 0.02, 95%CI = 0.01–0.04), and FAQ ($\beta$_standardized_ = 0.03, 95%CI = 0.01–0.04). After false-discovery rate correction, p-tau severity in the frontal, inferior parietal, and superior temporal cortex, and the amygdala was associated with higher CDS ($\beta$ s_standardized_ = 0.17–0.29, ps < 0.01) and FAQ ($\beta$ s_standardized_ = 0.21–0.26, ps < 0.01); frontal and inferior parietal cortex was associated with higher MI ($\beta$ s_standardized_ = 0.21–0.29, ps < 0.05); frontal cortex was associated with higher BRI ($\beta$_standardized_ = 0.21, *p* < 0.01). Regions with effects independent of other regions included frontal cortex (CDS, MI, FAQ, BRI), inferior parietal cortex (CDS) and amygdala (FAQ). P-tau explained 13–49% of variance in cognitive and functional scales and 6–14% of variance in neuropsychiatric scales.

**Conclusion:**

Accumulation of p-tau aggregates, especially in the frontal cortex, are associated with cognitive, functional, and certain neurobehavioral symptoms in CTE.

**Supplementary Information:**

The online version contains supplementary material available at 10.1186/s13024-023-00697-2.

## Background

Chronic traumatic encephalopathy (CTE) is a neurodegenerative disease caused in part by exposure to repetitive head impacts (RHI) from contact and collision sport participation (e.g., boxing, American football), physical violence, and other sources [1–3]. At this time, CTE can only be diagnosed at autopsy using published neuropathological diagnostic criteria [4, 5]. A neuropathological diagnosis of CTE requires the accumulation of perivascular hyper-phosphorylated tau (p-tau) in neurons with preference for the cerebral sulci [4, 5]. Four pathological stages of CTE severity have been defined [5–8]. In stage I CTE, 1 or 2 isolated foci of p-tau neurofibrillary tangles (NFTs) are found, typically in the frontal cortex. In stage II, p-tau lesions and superficial NFTs spread to adjacent temporal cortices. In stage III, NFTs are distributed in medial temporal lobe (MTL) structures. In stage IV, perivascular p-tau lesions and NFTs are distributed throughout the cerebral cortex, with pronounced neurofibrillary degeneration of the MTL.

The National Institute of Neurological Disorders and Stroke (NINDS) consensus diagnostic criteria for traumatic encephalopathy syndrome (TES) were published in 2021 [9]. TES is described as the clinical syndrome of CTE. The TES criteria are based, in part, on informant-reported symptoms of brain donors with autopsy-confirmed CTE [10, 11]. The TES criteria classify the core clinical features of CTE to include cognitive impairment (i.e., in episodic memory and/or executive function) and/or neurobehavioral dysregulation (e.g., impulsivity, short fuse). Cognitive and functional status (but not neurobehavioral dysregulation) and other supportive features are used to determine the level of certainty of underlying CTE pathology. Supportive features include psychiatric features (e.g., depression, apathy, anxiety, paranoia), motor signs and symptoms, and delayed symptom onset after RHI exposure. These TES criteria were developed to improve specificity compared with the original 2014 TES criteria [12]. However, the described clinical features among people with CTE pathology are heterogeneous and their etiology and association with CTE pathology require further study.

P-tau is a known precipitant of neurodegeneration and resulting cognitive and neuropsychiatric decline in Alzheimer’s disease and related dementias (AD/ADRD) [13–18]. In vivo tau positron emission tomography (PET) imaging studies in AD/ADRD show significant correspondence between regional tau PET binding and the different clinical manifestations of AD [18, 19]. Marshall et al. examined the association between regional flortaucipir PET and instrumental activities of daily living and apathy in 40 people with mild cognitive impairment (MCI) and AD dementia [20]. Findings suggest that functional impairment in AD might be related to tau burden in frontal and medial temporal lobe regions, whereas apathy is explained by tau in the right frontal cortex. In behavioral variant FTD (bvFTD), the presence of apathy and disinhibition hinge upon neurodegeneration in the dorsomedial and orbitofrontal lobe, respectively [21, 22]. A recent study of amyotrophic lateral sclerosis (ALS)-FTD showed that neurodegeneration of the dorsolateral prefrontal cortex corresponded to executive dysfunction; anterior cingulate cortex and dorsomedial prefrontal atrophy with apathy; and orbitofrontal atrophy with disinhibition [23].

We hypothesize similar associations in CTE. Published autopsy studies by our team have linked CTE p-tau severity with increased odds for antemortem dementia, based on informant-reported symptoms and judgment from a panel of expert clinicians [6, 24, 25]. Mez et al. evaluated the validity of the 2014 TES research diagnostic criteria and found that CTE p-tau pathology was associated with the presence of informant-reported cognitive but not behavioral or mood symptoms in 309 brain donors (244 with CTE) [11].

Detailed investigations on the association between CTE p-tau pathology and the various clinical features described in CTE have yet to be performed. The heterogeneity of clinical features observed in CTE might be explained by the regions that p-tau occupies, along with overall p-tau density. As in AD and frontotemporal dementia (FTD), p-tau aggregates in CTE affect neuroanatomical regions that modulate cognitive, mood, and behavior functions. For example, frontal p-tau aggregation is prominent in CTE and could contribute to executive dysfunction, neurobehavioral dysregulation, and apathy. Stage III and IV CTE involve p-tau aggregation in limbic system structures (e.g., hippocampus, entorhinal cortex, amygdala) known to modulate memory, mood, and behaviors.

Increased understanding of the association between CTE p-tau pathology and clinical symptoms will inform future clinical research diagnostic criteria and potential avenues for intervention. Here, we investigated the association between regional and density of CTE p-tau pathology across 10 brain regions and tested their associations with various cognitive, neuropsychiatric, and functional features described in the 2021 TES research diagnostic criteria.

## Methods

### Study design and brain donors

The sample included 364 deceased individuals who donated their brain to the Understanding Neurological Injury and Traumatic Encephalopathy (UNITE) brain bank as part of the UNITE research study [10]. The sample included only brain donors neuropathologically diagnosed with CTE. Most brain donations to the UNITE study originated with the next-of-kin contacting the brain bank near the time of death. Other brain donors were referred by medical examiners, recruited by a representative of the CLF, or participated in the Brain Donation Registry during life. Inclusion criterion for donation is having a history of exposure to RHI such as from contact and collision sport play, military service, physical violence, or other sources. Eligibility for brain bank enrollment is not determined by antemortem symptomatic status. Institutional review board (IRB) approval for brain donation, post-mortem clinical record review, interviews with informants, and neuropathological evaluation were obtained through the Boston University Medical Campus (BUMC) IRB. Informed consent for brain donation and study enrollment was obtained from the brain donors’ next-of-kin.

### Neuropathological diagnoses

Neuropathological evaluation occurred blinded to clinical data and was reviewed by study neuropathologists (VEA, BRH, TDS, ACM). Discrepancies in the neuropathological diagnosis were resolved by discussion and consensus of the group. Neuropathological processing and evaluation methods have been described [26, 27]. Brain weight and macroscopic features were recorded during initial processing. Twenty-two sections of paraffin-embedded tissue were stained for Luxol fast blue, hematoxylin and eosin (LHE), Bielschowsky’s silver, p-tau (AT8), alpha-synuclein, beta amyloid (Aß), and phosphorylated TDP-43 (pTDP-43). Neuropathological diagnosis of CTE was made using criteria defined by the NINDS-NIBIB Consensus Conference [4, 5]. The 2015 panel defined the pathognomonic lesion of CTE as “an accumulation of abnormal hyperphosphorylated tau (p-tau) in neurons and astroglia distributed around small blood vessels at the depths of cortical sulci and in an irregular pattern” [5]. This definition was refined in 2021 to require perivascular p-tau in neurons, with or without astrocytes, and the presence of at least one pathognomonic p-tau lesion in the cortex” [4]. The UNITE study has historically followed this criterion for the neuropathological diagnosis of CTE. CTE p-tau pathology was classified into four stages using the McKee staging criteria [6]. Established criteria were used for the neuropathological diagnosis of other neurodegenerative diseases, including AD (NIA-Reagan Institute criteria) [28], Lewy body disease (LBD) [29], frontotemporal lobar degeneration (FTLD) [26, 27, 30], and motor neuron disease (MND) [31]. Of note, CTE and primary age-related tauopathy (PART) can be difficult to differentiate due to overlap in regions affected. PART was determined using consensus criteria [32]. In participants meeting criteria for both CTE and PART, NFTs in CA4 were considered indicative of higher stage CTE; in the setting of advanced age and significant tangle burden in the entorhinal cortex, NFTs in CA1 were not considered indicative of higher stage CTE.

### Regional P-tau assessment

Assessments of the density of neuronal p-tau pathology were performed by the study neuropathologists at the time of initial diagnosis (blinded to all data with the exception of age) using semi-quantitative rating scales (0 = none, 1 = mild, 2 = moderate, 3 = severe). Semi-quantitative rating systems adhere to the National Alzheimer’s Coordinating Center (NACC) guidelines and recommendations [33]. These ratings are routine for all brain donors and allow for assessment of many cortical and subcortical regions throughout the brain. Our team of neuropathologists has been shown to have good inter-rater reliability using these rating scales [6]. AT8-immunostained, 10 µm thick paraffin-embedded sections of the following 10 regions were examined for this study (one section per stain per region): dorsolateral frontal cortex (DLFC), inferior frontal cortex (IFC), inferior parietal cortex (IPC), superior temporal cortex (STC), CA1-hippocampus, CA2-hippocampus, CA4-hippocampus, entorhinal cortex (EC), amygdala, and the locus coeruleus (LC, at the level of dorsal pons). Neuronal p-tau was assessed, and the entire gray matter was examined for cortical regions and the targeted subcortical structures. Subnuclei of the amygdala were not delineated. These regions were a priori selected because of their involvement in CTE [2, 5, 6, 8] and their known role in modulating cognitive, mood, and behavioral functions. Objectives of this study included examination of both the density and location of p-tau. As a single measure of overall p-tau density, we summed the rating scores across all 10 regions to form a global composite of p-tau severity (possible score range: 0—30). For the examination of regional p-tau, the DLFC and IFC were summed to form a frontal cortex composite (FC, possible score range: 0—6) and CA1, CA2, and CA4 were summed to create a hippocampal (HC) composite (possible score range: 0—9). The remaining regions (i.e., IPC, STC, EC, amygdala, and LC) were examined separately due to their more distinct neuroanatomical location.

### Informant interviews and standardized clinical scales

Methods and procedures for the retrospective evaluations of brain donors have been described in detail elsewhere [10, 24]. The evaluations of the brain donors were performed using online surveys and structured (e.g., questionnaires pertaining to cause of death, medical history, psychiatric history) and semi-structured (e.g., Ohio State University TBI Identification Method Short Form, modified Structured Clinical Interview for DSM) telephone interviews between researchers and informants of donors, all of whom were blinded to neuropathologic findings. On average, informants knew the donors for 42.55 (SD = 16.11) years. Medical record review is also completed. The evaluation is done in stages (five parts) and time to completion varies but efforts are made to complete all parts of the evaluation as soon as possible. Most pertinent to this study, the protocol involves the administration of standardized scales that assess cognitive function, neurobehavioral dysregulation, symptoms of depression and apathy, and daily function of the brain donors prior to death or at their worst (for those symptoms that tend to fluctuate over time, such as neuropsychiatric symptoms). All scales were modified to be appropriate for retrospective assessments by informants. The domains assessed and scales administered included:

#### Cognitive function

The Behavior Rating Inventory of Executive Function-Adult Version (BRIEF-A) Metacognition Index (MI) [34] and the Cognitive Difficulties Scale (CDS) [35] assessed informant-reported cognitive concerns. The BRIEF-A is a well-validated, 75 item measurement of executive function behaviors. Informants rated how often each behavior had been a problem on a three-point scale (1 = never, 2 = sometimes, 3 = often); higher scores indicate greater dysfunction. The BRIEF-A MI (40 items, range: 40—120) is a subscale of the BRIEF-A and reflects aspects of cognitive executive functions including activity initiation, problem-solving, working memory, planning, and organization.

The CDS is a 39-item instrument used to assess cognitive difficulties in attention, memory, perception, and psychomotor abilities [35]. Responses were made on a 5-point scale (0 = not at all, 4 = very often). Scores are summed for a global composite (range: 0—156) that served as an outcome; higher scores represent more cognitive concerns. Factor analyses on the CDS have been performed to derive sub-domain cognitive composites [36, 37]. However, there is scarce research on the CDS in an aging and dementia setting and none in a neurodegenerative disease brain bank. We therefore conducted a factor analysis on the CDS to derive factor scores that tap into different aspects of cognitive function (see Statistical Analyses section). In addition to the CDS total score, the derived CDS cognitive domain factor scores also served as outcomes.

#### Daily function

The Functional Activities Questionnaire (FAQ) is a 10-item scale of instrumental activities of daily living and scores range from 0 to 30, with higher scores reflecting greater severity of functional impairment [38]. A score of $\ge$ 9 is indicative of functional impairment.

#### Neurobehavioral dysregulation

Neurobehavioral dysregulation was assessed by the BRIEF-A BRI [34], Barratt Impulsiveness Scale (BIS-11) [39],and the Brown-Goodwin Aggression Scale [40]. The BRIEF-A BRI (30 items, range: 30—90) is another subscale of the BRIEF-A and reflects an individual’s ability to control impulses and self-monitor their behavior. The BIS-11 is a 30-item questionnaire designed to assess impulsiveness in three domains: motor, non-planning, and attention. BIS-11 items are ranked on a four-point Likert-type scale, where higher scores reflect more impulsive behaviors (range: 30—120). The Brown-Goodwin Aggression Scale is a 16-item assessment of lifetime (i.e., childhood, adolescence, adulthood) history of aggressive and impulsive behavior (verbal and/or physical). Informants rated the observed frequency of specific aggressive behaviors on a five-point scale (range: 11—44). A higher score indicates a higher frequency of aggressive behaviors. We only used the adulthood subsection of the Brown-Goodwin to reduce possibility of responses being reflective of lifelong and neurodevelopmentally-related aggression.

#### Depression and apathy

Symptoms of depression and apathy are supportive features of TES and were evaluated by the Geriatric Depression Scale 15-item version (GDS-15) [41, 42] and the Apathy Evaluation Scale (AES) [43], respectively. The GDS-15 is a 15-item yes/no checklist of depression symptoms (range: 0—15), with higher scores representing more severe symptoms of depression. The AES is an 18-item self-report measure of the cognitive, behavioral, and emotional symptoms of apathy. Informants ranked each symptom using a four-point Likert scale (1 = “not at all characteristic”, 4 = “very characteristic”; range: 18—72). Higher total AES scores indicate worse apathy.

### Demographic, athletic, RHI and TBI, and clinical history

Demographics, educational attainment, athletic history (type of sports played, level, position, age of first exposure and duration), military history, and traumatic brain injury history were queried during a telephone interview and/or using an online questionnaire. Medical records supplemented these sources. Here, we also specifically report on the presence of vascular risk factors and history of substance use due to their potential confounding of associations being investigated. Vascular risk factors included reported diagnostic history (absent/present) of hypertension and obstructive sleep apnea (OSA) [44, 45]. Informants were also asked if brain donors had ever received treatment for substance use and this served as an indicator of substance use severity as it is less influenced by informant recall.

### Statistical analyses

#### CDS factor analysis

A confirmatory factor analysis of the CDS was performed to derive domain-level cognitive factor scores. A clinical neuropsychologist (MLA) and behavioral neurologist (JM) assigned each item of the CDS to one of four cognitive domains, specifically attention, memory, language, and motor. These domain assignments were guided by previous factor analyses on the CDS [36, 37]. Based on the expert assignment, a multidimensional item response theory (MIRT) model was used to derive the CDS factor scores. We compared two models: nominal response model (NRM), which does not assume that response categories are ordered and can be used on nominal or ordinal scale data, and generalized partial credit model (GPCM), which is used when response categories may/may not be ordered and the categories may not have been used equally. Because the NRM had the smaller AIC, we used it as our final model and extracted the factor score estimates. Root mean square error of approximation (RMSEA), comparative fit index (CFI), and the Tucker Lewis Index (TLI) assessed model fit.

#### Analytic plan

Multivariable ordinary least squares (OLS) regression analyses examined the association between global p-tau severity and p-tau severity in each region with each of the clinical scales. The independent variables included the global and regional (i.e., FC, IPC, STC, HC, EC, amygdala, and LC) measures of p-tau severity, with each p-tau measure included in a separate model. The dependent variables included the clinical scales [i.e., cognitive/functional (BRIEF-A MI, CDS-total, CDS-attention, CDS-memory, CDS-language, CDS-motor, FAQ) and neuropsychiatric (BRIEF-A BRI, BIS-11, Brown-Goodwin-Adult, GDS-15, AES)] completed by the informants, with each scale or factor score included in a separate model. All models were adjusted for age at death, racial identity, education level, history of hypertension and OSA, and history of substance use treatment. Parameter estimates were standardized. For the regional p-tau models, false discovery rate (FDR) adjustment was based on seven analyses (i.e., seven regions) for each clinical scale. Sample sizes varied due to missing data across the standardized scales and model variables.

The above analyses did not account for correlation in p-tau pathology across regions. By accounting for this correlation, we could also investigate which regions may be driving the associations, independent of the effects from the other regions. Pairwise correlations among the regional measures of p-tau severity and age are presented in Supplemental Fig. 1 and showed a high correlation among the regions. To address concerns for multicollinearity and resulting unstable (large variance) parameter estimates, multivariable ridge regression was performed. Ridge regression is a parameter shrinkage approach used when the independent variables are highly correlated. Compared with OLS regression, ridge regression provides more precise parameter estimates due to smaller variances. Separate ridge regressions that included the seven regional p-tau measures and the same covariates as above were performed for each clinical scale/factor outcome.

To investigate how comorbid pathology may have impacted the observed relationships, we performed two sensitivity analyses: 1) Models were repeated with the Consortium to Establish a Registry for AD (CERAD) neuritic plaque score, limbic/neocortical LBD and FTLD added as additional covariates, and 2) Models were repeated after restricting the sample to donors without comorbid neurodegenerative disease, defined as AD, LBD, FTLD, and/or MND.

## Results

### Sample characteristics

Tables 1 and 2 present sample demographic, athletic, and neuropathology characteristics of the 364 brain donors with autopsy confirmed CTE. The sample was predominantly former American football players (333/364, 91.2%) who played at the college level or higher. Fifty-five (15.1%) self-reported being Black. One hundred forty (40.5%) had low (i.e., stage I and II) CTE. Two hundred thirty (63.2%) had CTE without other neurodegenerative disease diagnoses (i.e., CTE-only). The remaining sample had co-morbid neurodegenerative disease diagnoses, with the most common being AD. As shown in Table 1, 88.4% of the cases had none or sparse neuritic amyloid plaques.

**Table 1:** Sample Characteristics

	**Total Sample (*N* = 364)**
**Demographics**	
Age of death, mean (SD) years	62.48 (19.16)
Race, n (%) Black	55 (15.1)
Education Level, n (%)	
Some High School	2 (0.5)
High School Diploma/GED	10 (2.7)
Some College	72 (19.7)
College Degree	190 (52.2)
More than College	18 (4.9)
Graduate Degree	72 (19.8)
*APOE e4* carriers	78 (36.3)
**Athletics**	
Sport Played, n (%)^a^	
Football	333 (91.5)
Ice hockey	30 (8.2)
Wrestling	29 (8.0)
Soccer	28 (7.7)
Boxing	27 (7.4)
Skiing	2 (0.5)
Rugby	12 (3.3)
Lacrosse	10 (2.7)
Other	6 (1.6)
Years of Football Play, mean (SD)	13.71 (5.60)
Highest Level Football Played, n (%)	
Youth	5 (1.5)
High school	25 (7.5)
College	115 (34.5)
Semi-Professional	9 (2.7)
Professional	179 (53.8)
Military history, n (%)	89 (24.5)
**Medical Characteristics, n (%)**	
Hypertension	182 (50.8)
Obstructive sleep apnea	91 (25.8)
Substance use treatment	81 (22.4)
**Neuropathological Characteristics**	
CTE stage, n (%)	
Stage I	71 (19.5)
Stage II	69 (19.0)
Stage III	128 (35.2)
Stage IV	96 (26.4)
Alzheimer’s disease, n (%)	51 (14.0)
Braak Stage, n (%)	
0	75 (20.8)
I/II	89 (24.7)
III/IV	134 (37.1)
V/VI	63 (17.5)
CERAD neuritic plaque score, n (%)	
None	236 (64.8)
Sparse	86 (23.6)
Moderate	27 (7.4)
Severe	15 (4.1)
Thal Phase, n (%)	
0	162 (44.6)
1/2	54 (14.9)
3	49 (13.5)
4/5	98 (27.0)
Lewy body disease, n (%) neocortical	35 (9.6)
Frontotemporal lobar degeneration, n (%)	35 (9.7)
Motor neuron disease, n (%)	13 (3.6)
No comorbid neurodegenerative pathology	234

*Abbreviations:* *CTE* Chronic traumatic encephalopathy

^a^Categories are not mutually exclusive and there were 31 who did not play any American football

Missing data: *n *= 149 for *APOE;* *n* = 3 for highest level football played, frontotemporal lobar degeneration; *n* = 11 for obstructive sleep apnea; n = 6 for hypertension; *n* = 2 for substance use treatment; *n *= 1 for Thal phase

**Table 2:** Semi-quantitative ratings of p-tau severity, *n* (%)

**Dorsolateral frontal cortex, *n*** **= 360**	
None	30 (8.3)
Mild	103 (28.6)
Moderate	96 (26.7)
Severe	131 (36.4)
**Inferior frontal cortex, *n* = 352**	
None	79 (22.4)
Mild	122 (34.7)
Moderate	83 (23.6)
Severe	68 (19.3)
**Superior temporal cortex, *n* = 358**	
None	56 (15.6)
Mild	93 (26.0)
Moderate	100 (27.9)
Severe	109 (30.4)
**Inferior parietal cortex, *n *= 358**	
None	87 (24.3)
Mild	119 (33.2)
Moderate	63 (17.6)
Severe	89 (24.9)
**CA1-hippocampus, *n* = 356**	
None	72 (20.2)
Mild	124 (24.8)
Moderate	63 (17.7)
Severe	97 (27.2)
**CA2-hippocampus, *n* = 351**	
None	102 (29.1)
Mild	91 (25.9)
Moderate	86 (24.5)
Severe	72 (20.5)
**CA4-hippocampus, *n* = 355**	
None	93 (26.2)
Mild	133 (37.5)
Moderate	57 (16.1)
Severe	72 (20.3)
**Entorhinal cortex, *n* = 359**	
None	38 (10.6)
Mild	74 (20.6)
Moderate	94 (26.2)
Severe	153 (42.6)
**Amygdala, *n* = 358**	
None	47 (13.1)
Mild	74 (20.6)
Moderate	94 (26.2)
Severe	153 (42.6)
**Locus coeruleus, *n* = 337**	
None	23 (6.8)
Mild	72 (21.4)
Moderate	131 (38.9)
Severe	111 (32.9)

Sample sizes vary due to missing data. *Abbreviations*: *CTE* Chronic traumatic encephalopathy

**Fig. 1:**
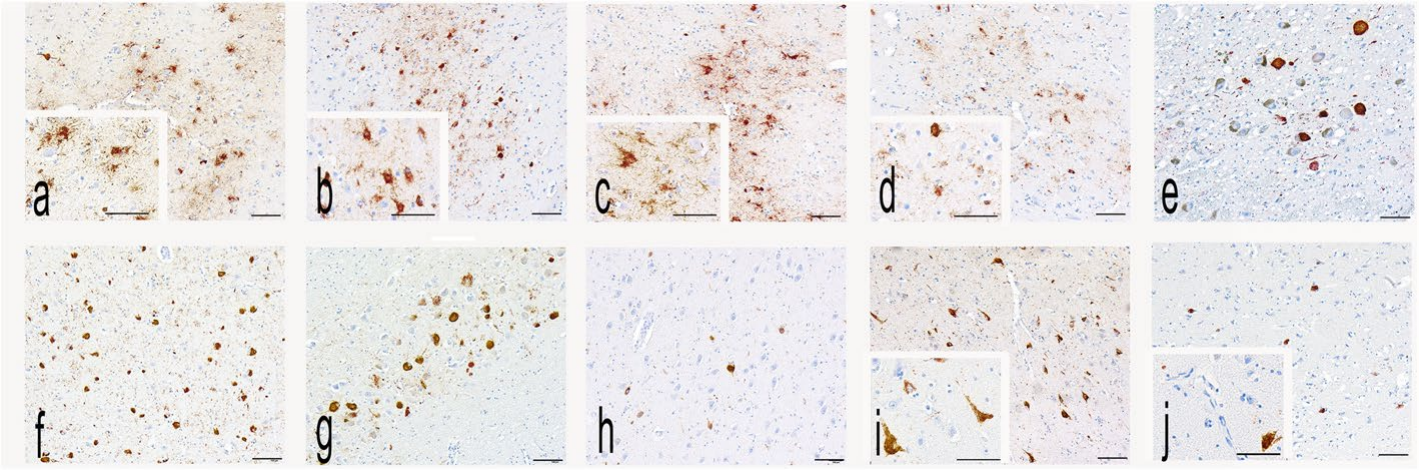
Regional Hyperphosphorylated Tau Pathology in CTE. Photomicrographs of AT8 immunostained cortical neurofibrillary tangles (NFTs), neurites, and astrocytic inclusions around small central vessels within the depths of sulci in a 71- year-old former professional American football player with stage IV CTE in the dorsolateral frontal cortex (**a**), inferior frontal cortex (**b**), inferior parietal cortex (**c**), superior temporal cortex (**d**), globose NFTs and neurities in the locus coeruleus (**e**), NFTs in the CA1 (f), CA2 (**g**) CA4 (**h**) hippocampal subfields, NFTs and neurites in the entorhinal cortex (**i**) layer 4 and basal nucleus of the amygdala (**j**). Scale bar = 100μm

Figure 1 shows representative images of p-tau pathology across the ten regions assessed in the study in a 71-year-old former professional American football player with severe (stage IV) CTE. Ratings of p-tau (Table 2) were most severe in MTL regions (particularly the amygdala and EC). Of the hippocampal subfields, CA1 was most severely affected, followed by CA2 and then CA4. The LC and DLFC were also among the regions most affected. Table 3 presents summary scores on the clinical scales for the full sample and stratified by CTE severity (i.e., stage I/II and stage III/IV) among those without comorbid neurodegenerative pathology. Impairment was common in cognitive function; 254 (75.8%) had elevated T-scores on the BRIEF-A MI (i.e., T-score ≥ 65) and 204 (58.6%) had an FAQ ≥ 9, considered meaningful functional impairment. There were high rates of elevated scores on the AES [265 (80.1%) had an AES > 34] and GDS-15 [265 (78.6%) had a GDS-15 score > 4].] Clinically meaningful symptoms of neurobehavioral dysregulation were present in 245 (73.1%; i.e., T-score ≥ 65 on the BRIEF-A BRI). Compared with donors with CTE stage I/II, donors with CTE stage III/IV had higher (worse) scores on cognitive and functional scales and similar scores on scales of behavioral dysregulation, depression and apathy.

**Table 3:** Descriptive statistics of informant-completed standardized clinical scales

	**Total Sample of Brain Donors with CTE ***(including donors with comorbid neurodegenerative pathology)*			**CTE Stage I/II**^a^*(excluding donors with comorbid neurodegenerative pathology)*			**CTE Stage III/IV**^a^*(excluding donors with comorbid neurodegenerative pathology)*		
	**Mean**	**SD**	**Impaired; n (%)**	**Mean**	**SD**	**Impaired; n (%)**	**Mean**	**SD**	**Impaired; n (%)**
**Cognitive Function**									
CDS, *n* = 336	92.99	47.32	–	64.24	36.25	–	91.73	43.25	–
BRIEF-A MI, *n* = 335	89.01	21.81	254 (75.8)	84.49	21.27	67 (65.7)	86.81	20.44	86 (76.8)
**Daily Function**									
FAQ, *n* = 348	14.73	11.57	204 (58.6)	5.80	7.13	24 (22.2)	15.52	11.09	76 (66.1)
**Neurobehavioral Dysregulation**									
BIS-11, *n* = 333	72.3	16.27	–	75.56	16.88	–	72.89	15.09	–
BRIEF-A BRI, *n* = 335	65.07	15.32	245 (73.1)	65.62	15.10	72 (70.6)	65.14	15.35	84 (75.0)
Brown-Goodwin-Adult Sum, *n* = 315	17.36	6.04	–	21.77	6.91	–	20.71	7.09	–
**Depression and Apathy**									
GDS-15, *n* = 337	8.81	4.41	265 (78.6)	9.41	4.76	78 (76.5)	8.58	4.17	91 (79.8)
AES, *n *= 331	48.40	14.31	265 (80.1)	44.15	14.23	71 (71.7)	48.54	13.25	93 (83.8)

*Abbreviations:* *CTE* Chronic traumatic encephalopathy, *CDS* Cognitive Difficulties Scale, *BRIEF-A* Behavior Rating Inventory of Executive Function for Adults, *MI* Metacognition Index, *FAQ* Functional Activities Questionnaire, *BIS*-11 Barratt Impulsiveness Scale, *GDS* Geriatric Depression Scale, 15-item version, *AES* Apathy Evaluation Scale, *AD* Alzheimer’s disease, *LBD* Lewy body disease, *FTLD *Frontotemporal lobar degeneration, *MND* Motor neuron disease

Note. Sample sizes vary across scales due to missing data and are based on the total sample. BRIEF-A MI and BRI are T-scores and a T ≥ 65 reflects clinically meaningful symptoms of executive dysfunction and behavioral dysregulation, respectively. A score of 9 or higher on the FAQ is indicative of functional impairment and scores of 5 and 34 or higher on the GDS-15 and AES represent clinically meaningful symptoms of depression and apathy, respectively. For the remaining scales, strongly supported cutoffs have not been identified in the literature

^a^Brain donors with comorbid neurodegenerative pathologies were excluded (i.e., AD, LBD, FTLD, MND)

Supplemental Table 1-3 present sample demographic, athletic, and neuropathological characteristics of the CTE-only group. Compared with the full sample, the CTE-only group was younger (mean difference = 17.2 years; *p* < 0.01), had less severe p-tau pathology, particularly in the cortical regions, and had lower (better) scores on scales of cognitive and daily function.

### CDS factor analysis

The factor analysis of the CDS using the NRM showed reasonable model fit (RMSEA = 0.11, TLI = 0.88, CFI = 0.91). Supplemental Fig. 2 shows the four factors and associated scale item loadings.

**Fig. 2:**
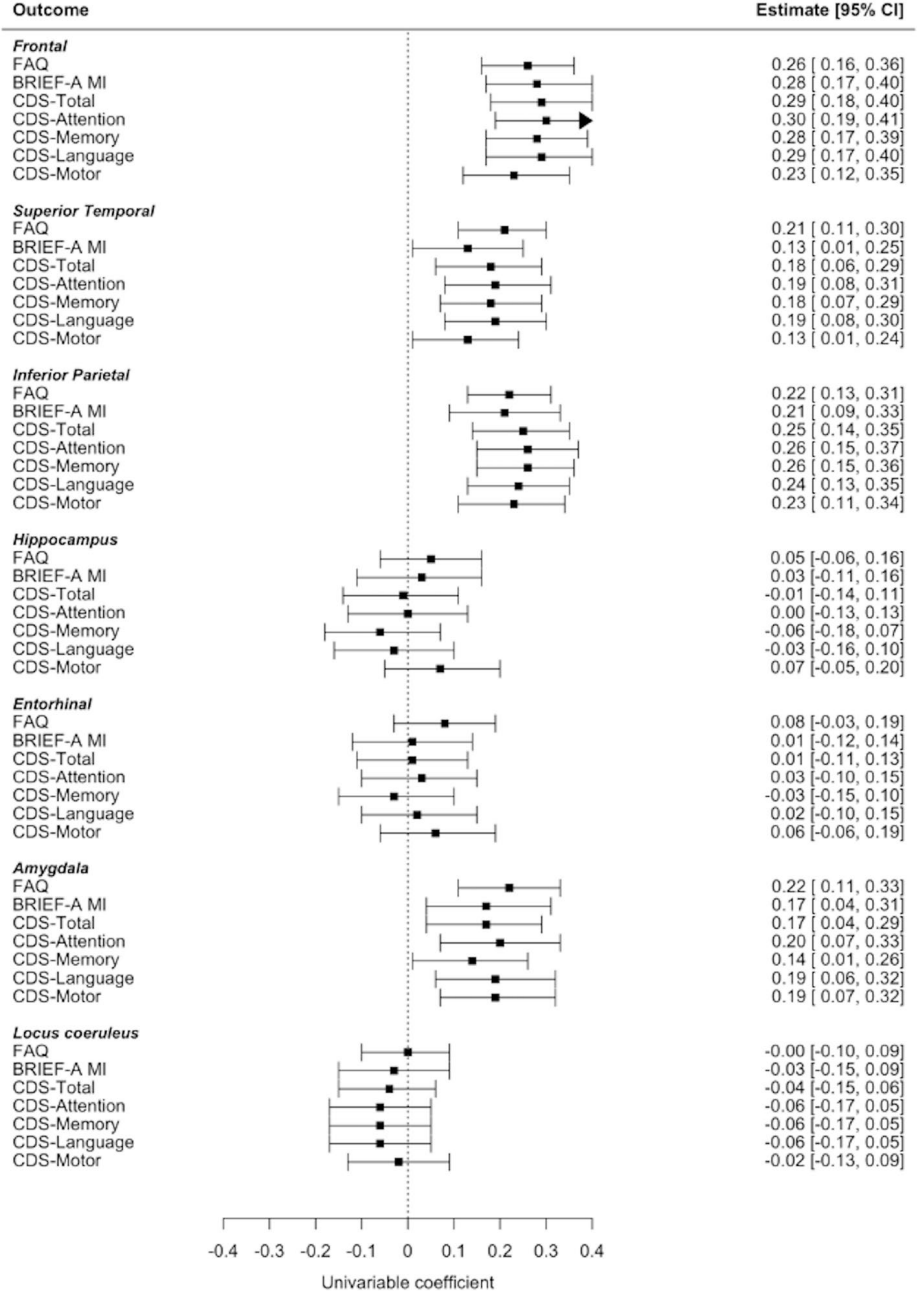
Forest Plot of The Estimated Effects of Regional P-tau Pathology on Standardized Cognitive Scales. Legend: Standardized estimates and associated 95% confidence intervals are from the multivariable OLS regression models (Table 4). The whiskers represent the 95% confidence intervals. Regional p-tau severity was rated on a 0–3 scale with 0 being none and 3 being severe. Frontal is a summary composite of the dorsolateral frontal cortex and the inferior frontal cortex. CA1, CA2, and CA4 were summed to create the hippocampus composite. For all clinical scales, higher scores are worse. Abbreviations: *OLS* Ordinary least squares, *CDS* Cognitive Difficulties Scale, *BRIEF-A* Behavior Rating Inventory of Executive Function for Adults, *MI* Metacognition Index, *FAQ* Functional Activities Questionnaire

### Investigating regions *individually* with multivariable OLS regression: associations between P-Tau severity and standardized clinical scales

**Fig. 3:**
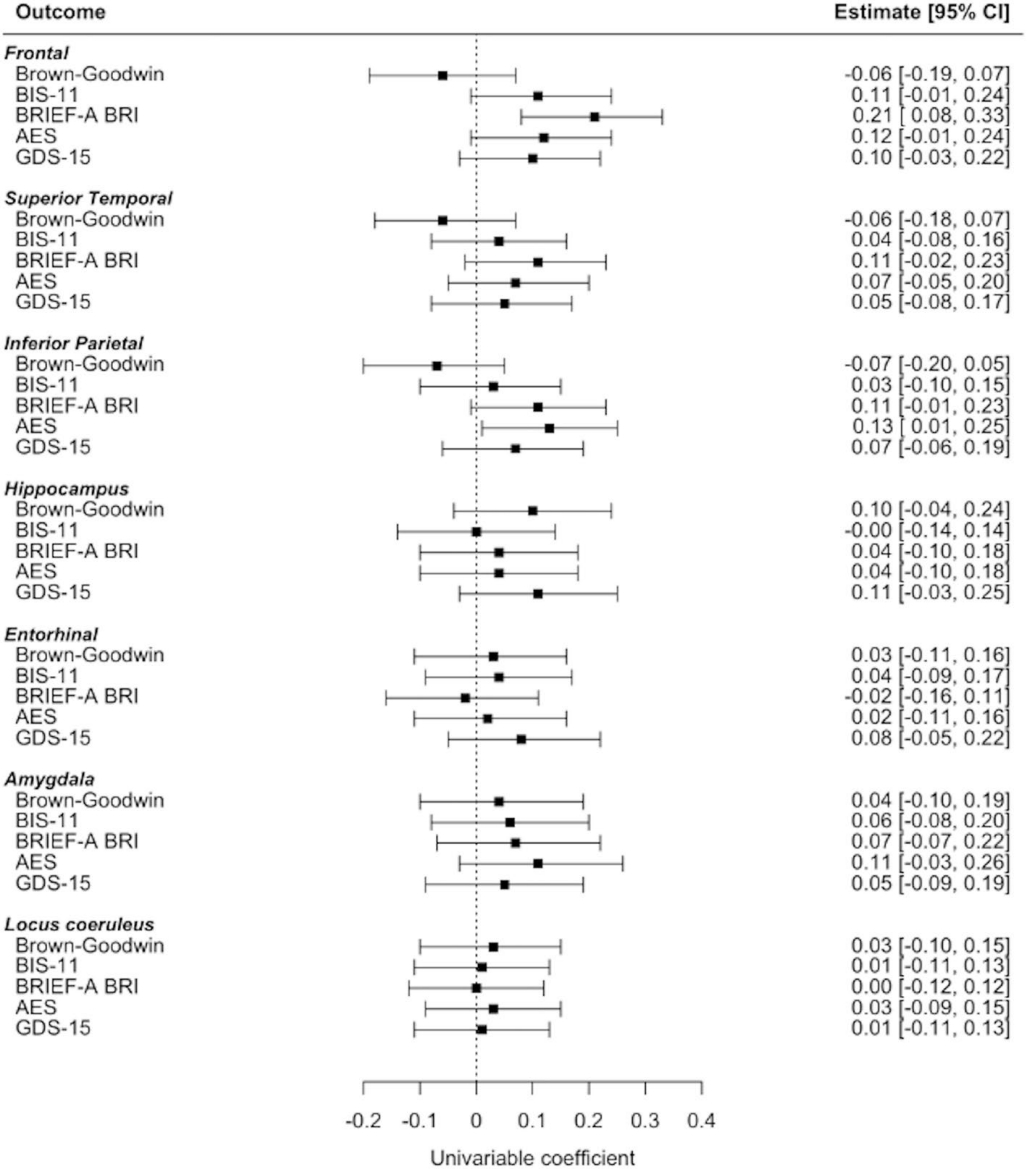
Forest Plot of The Estimated Effects of Regional P-tau Pathology on Standardized Scales of Neurobehavioral Dysregulation, Depression, and Apathy. Legend: Standardized estimates and associated 95% confidence intervals are from the multivariable OLS regression models (Table 4). The whiskers represent the 95% confidence intervals. Regional p-tau severity was rated on a 0–3 scale with 0 being none and 3 being severe. Frontal is a summary composite of the dorsolateral frontal cortex and the inferior frontal cortex. CA1, CA2, and CA4 were summed to create the hippocampus composite. For all clinical scales, higher scores are worse. Abbreviations: *OLS* Ordinary least squares, *BRIEF-A* Behavior Rating Inventory of Executive Function for Adults, *BRI* Behavioral Regulation Index, *BIS-11 *Barratt Impulsiveness Scale, *GDS* Geriatric Depression Scale, 15-item version, *AES* Apathy Evaluation Scale

A summary of regression models are provided in Tables 4 and 5. Figures 2 and 3 present forest plots of the estimated effects between regional p-tau pathology and the clinical scales. Below, we summarize the results by regional location of p-tau.

**Table 4:** Association between regional p-tau and cognitive and daily function scales: summary of multivariable OLS regression results

	**CDS Total Score** (*n* = 284)			**CDS Attention Factor Score** (*n* = 284)			**CDS Memory Factor Score** (*n *= 284)			**CDS Language Factor Score** (*n *= 284)			**CDS Motor Factor Score** (*n* = 284)			**BRIEF-A MI** (*n* = 285)			**FAQ** (*n* = 290)		
**P-tau ratings**	**b**	**95% CI**	**p**_**adj**_	**b**	**95% CI**	**p**_**adj**_	**b**	**95% CI**	**p**_**adj**_	**b**	**95% CI**	**p**_**adj**_	**b**	**95% CI**	**p**_**adj**_	**b**	**95% CI**	**p**_**adj**_	**b**	**95% CI**	**p**_**adj**_
Frontal	0.29	0.18, 0.4	< 0.01	0.3	0.19, 0.41	< 0.01	0.28	0.17, 0.39	< 0.01	0.29	0.17, 0.40	< 0.01	0.23	0.12, 0.35	< 0.01	0.29	0.17, 0.42	< 0.01	0.26	0.16, 0.36	< 0.01
Superior temporal	0.18	0.06, 0.29	< 0.01	0.19	0.08, 0.31	< 0.01	0.18	0.07, 0.29	< 0.01	0.19	0.08, 0.30	< 0.01	0.13	0.01, 0.24	0.09	0.13	0.00, 0.26	0.12	0.21	0.11, 0.30	< 0.01
Inferior parietal	0.25	0.14, 0.35	< 0.01	0.26	0.15, 0.37	< 0.01	0.26	0.15, 0.36	< 0.01	0.24	0.13, 0.35	< 0.01	0.23	0.11, 0.34	< 0.01	0.21	0.08, 0.33	< 0.01	0.22	0.13, 0.31	< 0.01
Amygdala	0.17	0.04, 0.29	0.03	0.2	0.07, 0.33	< 0.01	0.14	0.01, 0.26	0.12	0.19	0.06, 0.32	< 0.01	0.19	0.07, 0.32	< 0.01	0.17	0.03, 0.32	0.07	0.22	0.11, 0.33	< 0.01
Entorhinal	0.01	-0.11, 0.13	0.90	0.03	-0.10, 0.15	0.74	-0.03	-0.15, 0.1	0.90	0.02	-0.1, 0.15	0.80	0.06	-0.06, 0.19	0.58	0.01	-0.13, 0.15	0.92	0.08	-0.03, 0.19	0.34
Hippocampus	-0.01	-0.14, 0.11	0.90	0.00	-0.13, 0.13	0.99	-0.06	-0.18, 0.07	0.84	-0.03	-0.16, 0.1	0.80	0.07	-0.05, 0.20	0.54	0.03	-0.11, 0.17	0.92	0.05	-0.06, 0.16	0.68
*Locus coereulus*	-0.04	-0.15, 0.06	0.81	-0.06	-0.17, 0.05	0.74	-0.06	-0.17, 0.05	0.72	-0.06	-0.17, 0.05	0.56	-0.02	-0.13, 0.09	0.85	-0.02	-0.14, 0.10	0.92	0.00	-0.1, 0.09	0.99

*Abbreviations*: *OLS* Ordinary least squares, *CDS* Cognitive Difficulties Scale, *BRIEF-A* Behavior Rating Inventory of Executive Function for Adults, *MI* Metacognition Index, *FAQ* Functional Activities Questionnaire

Estimates are standardized betas. Regional p-tau severity was rated on a 0–3 scale with 0 being none and 3 being severe. Frontal is a summary composite of the dorsolateral frontal cortex and the inferior frontal cortex. CA1, CA2, and CA4 were summed to create the hippocampus composite. Each region was examined without other regions in the model. For all clinical scales, higher scores are worse. P-values were false discovery rate adjusted using the Benjamini–Hochberg procedure. Models were adjusted for age at death, racial identity, education level, history of hypertension and obstructive sleep apnea, and history of substance use treatment

**Table 5:** Association between regional p-tau and scales of neurobehavioral dysregulation, depression, and apathy: summary of multivariable OLS regression results

	**BRIEF-A BRI** (*n* = 285)			**BIS-11** (*n* = 283)			**Brown Goodwin** (*n *= 263)			**GDS-15** (*n* = 286)			**AES** (*n* = 281)		
**P-tau ratings**	**b**	**95% CI**	**p**_**adj**_	**b**	**95% CI**	**p**_**adj**_	**b**	**95% CI**	**p**_**adj**_	**b**	**95% CI**	**p**_**adj**_	**b**	**95% CI**	**p**_**adj**_
Frontal	0.21	0.08, 0.34	< 0.01	0.11	-0.01, 0.24	0.35	-0.06	-0.19, 0.07	0.53	0.10	-0.03, 0.22	0.4	0.12	-0.01, 0.24	0.35
Superior temporal	0.10	-0.02, 0.23	0.33	0.04	-0.08, 0.16	0.94	-0.06	-0.18, 0.07	0.53	0.05	-0.08, 0.17	0.55	0.07	-0.05, 0.2	0.56
Inferior parietal	0.10	-0.02, 0.23	0.33	0.03	-0.1, 0.15	0.94	-0.07	-0.2, 0.05	0.43	0.07	-0.06, 0.19	0.40	0.13	0.01, 0.25	0.30
Amygdala	0.07	-0.08, 0.21	0.75	0.06	-0.08, 0.2	0.94	0.04	-0.1, 0.19	0.70	0.05	-0.09, 0.19	0.55	0.11	-0.03, 0.26	0.41
Entorhinal	-0.03	-0.16, 0.11	0.91	0.04	-0.09, 0.17	0.94	0.03	-0.11, 0.16	0.72	0.08	-0.05, 0.22	0.40	0.02	-0.11, 0.16	0.76
Hippocampus	0.04	-0.10, 0.18	0.91	< 0.01	-0.14, 0.14	0.97	0.10	-0.04, 0.24	0.34	0.11	-0.03, 0.25	0.40	0.04	-0.1, 0.18	0.76
Locus coereulus	0.01	-0.11, 0.14	0.91	0.01	-0.11, 0.13	0.97	0.03	-0.1, 0.15	0.72	0.01	-0.11, 0.13	0.88	0.03	-0.09, 0.15	0.76

*Abbreviations*: *OLS* Ordinary least squares, *BRIEF-A* Behavior Rating Inventory of Executive Function for Adults, *BRI* Behavioral Regulation Index, *BIS-11* Barratt Impulsiveness Scale, *GDS* Geriatric Depression Scale, 15-item version, *AES* Apathy Evaluation Scale

Estimates are standardized betas. Regional p-tau severity was rated on a 0–3 scale with 0 being none and 3 being severe. Frontal is a summary composite of the dorsolateral frontal cortex and the inferior frontal cortex. CA1, CA2, and CA4 were summed to create the hippocampus composite. Each region was examined without other regions in the model. For all clinical scales, higher scores are worse. P-values were false discovery rate adjusted using the Benjamini–Hochberg procedure. Models were adjusted for age at death, racial identity, education level, history of hypertension and obstructive sleep apnea, and history of substance use treatment

#### Global P-tau severity

Multivariable linear regressions adjusted for age, race, education level, hypertension, OSA, and substance use treatment showed statistically significant associations between the global p-tau severity composite and scales of cognitive function and daily function: BRIEF-A MI (standardized beta = 0.02, 95% CI = 0.01–0.04, *p* = 0.01), CDS total score (standardized beta = 0.02, 95% CI = 0.01–0.04, *p* = 0.01), CDS-Attention (standardized beta = 0.03, 95% CI = 0.01–0.04, *p* < 0.01), CDS-Memory (standardized beta = 0.02, 95% CI = 0.00–0.04, *p* = 0.02), CDS-Language (standardized beta = 0.02, 95% CI = 0.01–0.0404, *p* = 0.01), CDS-Motor (standardized beta = 0.03, 95% CI = 0.01–0.04, *p *< 0.01), and FAQ (standardized beta = 0.03, 95% CI = 0.01–0.04, *p* < 0.01). Greater severity of p-tau was associated with worse cognitive and daily function. The global p-tau severity composite was not associated with the neuropsychiatric scales (ps > 0.10 for all).

#### Frontal cortex

P-tau severity in the FC was significantly associated with all cognitive (i.e., BRIEF-A MI, CDS total, CDS-Attention, CDS-Memory, CDS-Language, CDS-Motor) and daily function (i.e., FAQ) scales (standardized beta range: 0.23–0.30; ps_adj_ < 0.01). Among the neuropsychiatric scales, FC p-tau severity was associated with the BRIEF-A BRI (standardized beta: 0.21; p_adj_ < 0.01), a measure of behavioral dysregulation. For all associations, greater p-tau severity corresponded to higher (i.e., worse) scores on the scales. FC p-tau severity was not associated with the AES, Brown-Goodwin, GDS-15, or BIS-11 (ps > 0.05).

#### Superior temporal cortex

Greater p-tau severity in the STC corresponded to higher (i.e., worse) scores on measures of cognitive and daily function, including the CDS total, CDS-Attention, CDS-Memory, CDS-Language, and FAQ (standardized beta range: 0.18–0.21, ps_adj_ < 0.01 for all). No other significant associations were noted for this region.

#### Inferior parietal cortex

IPC p-tau severity was significantly associated with higher (i.e., worse) scores on all cognitive and functional scales (standardized beta range: 0.21–0.26, ps_adj_ < 0.01). The IPC was not associated with any of the neuropsychiatric scales (ps > 0.10).

#### Entorhinal cortex

EC p-tau severity was not associated with any of the clinical scales (ps > 0.10).

#### Amygdala

Greater p-tau severity in the amygdala was associated with higher (i.e., worse) scores on the cognitive and functional scales, including CDS total score, CDS-Attention, CDS-Language, CDS-Motor, and the FAQ (standardized beta range: 0.17–0.22, ps_adj_ < 0.05). Amygdala p-tau severity was not associated with any of remaining clinical scales (ps > 0.10 for all).

#### Hippocampus

P-tau severity in the hippocampus was not associated with any of the clinical scales (ps > 0.10 for all).

#### Locus coeruleus

P-tau severity in the locus coeruleus was not associated with any of the clinical scales (ps > 0.10 for all).

### Investigating regions *jointly* with multivariable ridge regression: associations between P-Tau severity and standardized clinical scales

Ridge regression models were performed to identify which regions had p-tau pathology associated with the clinical scales, independent of the other regions. Ridge regression reduces concerns about multicollinearity between regions (Supplemental Fig. 1). Results are summarized in Table 6. P-tau pathology in the FC showed significant associations across all scales of cognitive function (standardized beta range: 0.14–0.24), daily function (FAQ; standardized beta: 0.16), and behavioral dysregulation (BRIEF-A BRI; standardized beta: 0.11). P-tau pathology in the amygdala and IPC were the only other regions that continued to be significantly associated with any of the scales. Specifically, amygdala p-tau pathology was significantly associated with the FAQ (standardized beta: 0.13). IPC p-tau pathology was significantly associated with CDS total, CDS-Attention, CDS-Memory, CDS-language and CDS-Motor (standardized beta range: 0.11–0.16). The amount of the variance in the scales explained (R^2^) by the full ridge regression models differed markedly (from largest to smallest): FAQ: 0.49; CDS total: 0.36; CDS memory: 0.36; CDS language: 0.34; CDS attention: 0.33; CDS motor 0.30; Brown-Goodwin: 0.14; BIS: 0.14; BRIEF-A MI: 0.13; AES: 0.09; BRIEF-A BRI: 0.08; GDS: 0.06.

**Table 6:** Association between regional p-tau and standardized scales: summary of ridge regression results

	**CDS Total**		**CDS-Attention**		**CDS. Memory**		**CDS Language**		**CDS Motor**		**BRIEF-A MI**		**FAQ**		**BRIEF-A BRI**		**BIS-11**		**Brown-Goodwin**		**GDS-15**		**AES**	
	**Ridge coef**	**95% CI**	**Ridge coef**	**95% CI**	**Ridge coef**	**95% CI**	**Ridge coef**	**95% CI**	**Ridge coef**	**95% CI**	**Ridge coef**	**95% CI**	**Ridge coef**	**95% CI**	**Ridge coef**	**95% CI**	**Ridge coef**	**95% CI**	**Ridge coef**	**95% CI**	**Ridge coef**	**95% CI**	**Ridge coef**	**95% CI**
Frontal	0.24	0.14, 0.37	0.22	0.13, 0.34	0.23	0.11, 0.35	0.22	0.12, 0.36	0.14	0.07, 0.24	0.19	0.10, 0.29	0.16	0.07, 0.29	0.11	0.05, 0.18	0.07	0.00, 0.15	-0.04	-0.10, 0.03	0.02	-0.03, 0.07	0.04	-0.01, 0.10
Superior temporal	0.00	-0.13, 0.11	0.01	-0.11, 0.11	0.02	-0.10, 0.13	0.02	-0.11, 0.13	-0.02	-0.11, 0.07	-0.01	-0.09, 0.07	0.06	-0.02, 0.17	0.02	-0.04, 0.09	-0.01	-0.09, 0.06	-0.04	-0.10, 0.04	0.00	-0.05, 0.05	0.01	-0.05, 0.05
Inferior parietal	0.13	0.02, 0.24	0.13	0.02, 0.24	0.16	0.06, 0.27	0.11	0.01, 0.21	0.12	0.03, 0.21	0.07	-0.02, 0.15	0.08	-0.03, 0.18	0.01	-0.06, 0.08	-0.02	-0.07, 0.05	-0.05	-0.12, 0.01	0.00	-0.06, 0.05	0.05	-0.01, 0.11
Amygdala	0.08	-0.04, 0.2	0.10	-0.02, 0.21	0.06	-0.08, 0.19	0.12	0.00, 0.23	0.10	0.00, 0.19	0.06	-0.04, 0.15	0.13	0.02, 0.23	-0.01	-0.08, 0.05	0.01	-0.06, 0.09	0.01	-0.07, 0.07	-0.02	-0.06, 0.03	0.05	0.00, 0.10
Entorhinal	-0.10	-0.22, 0.00	-0.09	-0.21, 0.00	-0.13	-0.25, -0.02	-0.07	-0.19, 0.03	-0.03	-0.13, 0.06	-0.07	-0.17, 0.01	-0.05	-0.16, 0.06	-0.06	-0.13, 0.00	0.00	-0.08, 0.07	0.00	-0.06, 0.07	0.01	-0.05, 0.07	-0.01	-0.07, 0.04
Hippocampus	-0.05	-0.17, 0.06	-0.04	-0.15, 0.07	-0.07	-0.19, 0.05	-0.07	-0.18, 0.05	0.03	-0.07, 0.13	0.00	-0.09, 0.09	-0.03	-0.14, 0.06	0.00	-0.07, 0.07	-0.04	-0.11, 0.02	0.04	-0.03, 0.11	0.02	-0.03, 0.08	0.02	-0.03, 0.08
Locus coeruleus	-0.09	-0.18, 0.00	-0.12	-0.22, -0.04	-0.10	-0.2, -0.02	-0.11	-0.2, -0.01	-0.08	-0.18, 0.00	-0.05	-0.13, 0.03	-0.07	-0.17, 0.01	0.00	-0.08, 0.08	0.00	-0.07, 0.08	0.01	-0.07, 0.10	0.00	-0.06, 0.07	0.01	-0.05, 0.07

*Abbreviations*: *CDS* Cognitive Difficulties Scale, *BRIEF-A* Behavior Rating Inventory of Executive Function for Adults, *MI* Metacognition Index, *FAQ* Functional Activities Questionnaire, *BRI* Behavioral Regulation Index, *BIS-11* Barratt Impulsiveness Scale, *GDS* Geriatric Depression Scale, 15-item version, *AES* Apathy Evaluation Scale

To investigate which regions may be driving the associations, independent of the effects from the other regions, multivariable ridge regression models were performed for the regional scales of p-tau severity. Ridge regression is used when there are many independent variables that are highly correlated. A separate ridge regression for each regional scale of p-tau severity was performed for each clinical scale. Estimates are standardized betas. Models were adjusted for age at death, racial identity, education level, history of hypertension and obstructive sleep apnea, and history of substance use treatment. Frontal is a summary composite of the dorsolateral frontal cortex and the inferior frontal cortex. CA1, CA2, and CA4 were summed to create the hippocampus composite. For all clinical scales, higher scores are worse. As shown, the frontal cortex showed independent effects in the prediction of scales. However, the inferior parietal cortex (CDS) and amygdala (FAQ, AES) showed smaller independent effects

### Sensitivity analyses: role of comorbid neurodegenerative pathology

Supplemental Table 4 and 5 summarize the results from the OLS regression analyses separately investigating the relationship between p-tau pathology in each region and standardized scales, after adding the CERAD neuritic plaque score, limbic/neocortical LBD and FTLD as covariates. Supplemental Table 6 summarizes the results from ridge regression jointly investigating the relationship between p-tau pathology in each region and standardized scales, after adding the CERAD neuritic plaque score, limbic/neocortical LBD and FTLD as covariates. In general, estimated effect sizes were reduced, but associations between frontal p-tau and cognitive and functional scales remained significant. Supplemental Table 7 and 8 summarize the results from the OLS regression analyses separately investigating the relationship between p-tau pathology in each region and standardized scales, after restricting the sample to donors without comorbid neurodegenerative disease. Supplemental Table 9 summarizes the results from ridge regression jointly investigating the relationship between p-tau pathology in each region and standardized scales, after restricting the sample to donors without comorbid neurodegenerative disease. In general, as with the sensitivity models adjusting for comorbid neurodegenerative pathology, estimated effect sizes were reduced compared with the primary models, with the largest effect sizes remaining for p-tau pathology in the frontal lobe and cognitive outcomes. Both sensitivity models had similar effect sizes, but effects only remained significant for models adjusted for comorbid neurodegenerative pathology. This is expected given the smaller sample size when donors with comorbid neurodegenerative pathology (n≈100) were removed; we were only ~ 40% powered to detect significant associations for the largest observed effect size with the smaller sample size.

## Discussion

In this sample of 364 brain donors with autopsy confirmed CTE, we examined the contribution of global and regional p-tau density to informant-reported cognitive, functional, and neuropsychiatric symptoms (including symptoms of neurobehavioral dysregulation, depression and apathy). Global p-tau severity was associated with greater reported cognitive difficulties, including in executive functions, as well as greater daily functional difficulties. Among the seven different cortical and subcortical regions examined, p-tau density in the FC was the strongest contributor to cognitive and functional symptoms, but there was also contribution from the IPC and amygdala. As opposed to global p-tau density, the location of p-tau, particularly in the FC, was most important for explaining symptoms of neurobehavioral dysregulation. In general, however, there were weaker effects for scales that assessed neuropsychiatric symptoms. These findings suggest that in CTE, accumulation of p-tau aggregates, especially in the FC, is associated with cognitive and function symptoms, as well as certain symptoms of neurobehavioral dysregulation.

Impairments in memory and executive function are core clinical features of the NINDS consensus diagnostic criteria for the clinical syndrome of CTE known as TES [9]. Functional impairments are also part of the TES criteria and are used for determining level of CTE certainty (i.e., suggestive, possible, probable). The specificity of these cognitive and functional features to CTE are poorly understood in part due to the lack of studies directly relating them to CTE p-tau pathology [11, 24]. In the present study, p-tau severity was associated with reported cognitive and functional symptoms, with models explaining 13 to 49% of the variance in standardized scales. The strongest contribution came from the FC, followed by the IPC and amygdala. Although STC p-tau pathology was associated with cognitive and functional symptoms in regression models that did not include other regions, it was not significantly associated in the ridge regressions that investigated which regions showed independent effects. Our findings are in concordance with research in AD/ADRD that similarly show impairments in cognitive and behavioral functions that correspond with brain regions targeted by the disease [13, 18]. There is ample evidence suggesting that executive dysfunction, driven by FC pathology, is the primary cognitive domain associated with functional decline in AD [46, 47]. In CTE, the FC is the initial location of p-tau aggregation and becomes heavily affected with disease progression [6]. Over time, adjacent regions such as the temporal lobe and IPC become involved. The MTL, including the amygdala, is affected in high stage CTE (i.e., stage III and IVIV) [6, 7].*In vivo*evidence increasingly shows a frontotemporal pattern of atrophy in CTE, as well as tau tracer uptake on PET in the FC [48–52]. Given the known function of the FC and the involvement of the FC in CTE, the FC association with cognitive (particularly executive) and functional symptoms was expected. The observed contributions of p-tau in the IPC and amygdala to cognitive difficulties in this study are also noteworthy. Interestingly, Vogel et al [53] showed that a parietal-dominant and MTL-sparing subtype of AD had more overall tau burden and worse relative executive function compared to other identified AD subtypes. Although the amygdala is typically known to modulate anxiety [54, 55] and aggression [56] in AD, the association between p-tau in the amygdala and reported functional impairment aligns with past research that showed the degree of amygdala atrophy in AD is associated with severity of cognitive impairment [54].Our findings highlight p-tau pathology in the amygdala as another potential contributing factor to cognitive symptoms in TES. In sum, global and regional p-tau, particularly in the FC, are likely key determinants of cognitive and functional difficulties in CTE.

Neurobehavioral dysregulation is also a core feature of the TES criteria [8, 9, 12]. Unlike cognitive difficulties, the pathological underpinnings of neurobehavioral dysregulation in CTE have been elusive. For example, Mez et al. found CTE p-tau pathology was associated with the presence of informant-reported cognitive, but not behavioral or mood symptoms [11]. Other studies have suggested non-tau causes of symptoms of neurobehavioral dysregulation in CTE [57], including the possibility that these are lifelong as the populations being studied are preselected to be more aggressive and impulsive [58]. For these reasons, the 2021 TES criteria consider cognitive impairment, but not neurobehavioral dysregulation, to determine the provisional levels of certainty for CTE pathology. Indeed, in the current study, the ridge regression models that incorporated p-tau pathology measures across brain regions, only explained 8–14% of the variance in the scales measuring behavioral dysregulation suggesting there are other explanatory factors not being captured in the models. Like the Mez et al. study, global p-tau burden did not explain symptoms of neurobehavioral dysregulation. Instead, p-tau deposition in the FC was associated with higher scores on the BRIEF-BRI, a measure of one’s ability to regulate emotions and behavior [34]. The FC is responsible for executive processes like self-monitoring which if disrupted from lesions or degenerative processes can result in impulsivity and disinhibition [59–61]. Previous studies have supported the role of the FC in behavioral dysregulation. For example, volume loss in the FC has been linked to disinhibition in people with AD [60] and FTD [62]. Notably, the FC was not associated with other scales of neurobehavioral dysregulation. This could be related to poor measurement or that each scale may capture a different aspect of neurobehavioral dysregulation. The BRIEF-A BRI has been shown to capture symptoms associated with exposure to RHI [63, 64]. Our findings suggest that p-tau in the FC might partially contribute to aspects of neurobehavioral dysregulation in CTE. Additionally, the current study may suggest that scales that better capture the specific symptoms of neurobehavioral dysregulation observed in CTE should be developed.

We also conducted sensitivity analyses to investigate how comorbid pathology may be contributing to the observed clinicopathological associations. When we adjusted for CERAD neuritic plaque score, limbic/neocortical LBD and FTLD, effect sizes were reduced, but remained significant for most of the previously significant associations, suggesting that the observed associations were at least in part independent of comorbid neurodegenerative pathology. Restricting the sample to donors without comorbid neurodegenerative pathology resulted in similar effect sizes as adjusting for comorbid neurodegenerative pathology. Although the associations were no longer significant, the sample size was reduced by more than a third and the remaining sample had a considerably smaller burden of p-tau pathology. While isolating CTE pathology allows for increased understanding of disease specific effects, most neurodegenerative diseases do not occur in isolation and symptoms are often a result of mixed pathologies [65]. This might be particularly true for CTE given exposure to RHI has been associated with various neuropathologies [24, 66, 67].

Several regions had no associations with the scales of cognitive function, daily function, and neuropsychiatric symptoms, including the EC, hippocampus, and LC. Aspects of these findings conflict with existing research in AD. For example, p-tau accumulation in regions of the MTL has been associated with episodic memory decline [62] in addition to functional impairment in AD [68]. Degeneration of the LC has also been associated with impaired memory and attention [69, 70]. In concordance with our findings, maintenance of impulse control has also been associated with preservation of the LC-norepinephrine system independent of tau pathology in AD [71].An additional unexpected finding was the lack of effects between amygdala p-tau severity and neurobehavioral scores despite the known association between amygdala neurodegeneration and agitation and aggression in AD [56]. While several of these regions are severely affected in CTE, it is not until high stage. Additionally, limited assessment of certain sub-domains, such as episodic memory, that may be hard to assess with informant reported scales, might explain the lack of associations for certain regions (e.g., hippocampus). Also, recent evidence suggests that p-tau pathology and hippocampal volume may have larger effects on cognition in the “permissive” state of having amyloid pathology present [72, 73]. Further, the sample was highly symptomatic, and restriction of scale range could have contributed to null findings. In general, null findings should be interpreted with caution. Although this study benefited from gold standard pathology, it lacked prospective, objective clinical assessments, introducing potential measurement error and recall bias. It is also possible that null findings in the setting of symptomatic donors may occur because other types of pathologies associated with RHI, such as axonal and myelin injury, might account for symptoms [24, 74–76]. Indeed, even for the ridge regression models that explained the most variance in the clinical scales, > 50% of the variance remained unexplained, suggesting other pathologies may be contributing. This is a target of separate ongoing investigations.

In addition to sub-optimal measurement of clinical symptoms, the study has other limitations. Semi-quantitative scales were used to rate p-tau severity as opposed to quantitative methods in order maximize regional coverage of the brain and minimize missing data. Quantitative measurement of p-tau density is ongoing at our Center, but is time intensive and currently only available for a few regions. It is possible that semi-quantitative scales have more measurement error which could explain null findings, such as the lack of association between hippocampal tau and cognition. The regions examined were selected a priori due to their known involvement in CTE and roles in modulating neurobehavioral function. We acknowledge that we did not include all hippocampal subfields (i.e., CA3), nor did we delineate subnuclei of the amygdala or assess other forms of tau (e.g., oligomeric and astrocytic tau). These may be additional explanations for some of the null findings. The generalizability of the results may be limited due to ascertainment bias associated with brain donation. Donors with CTE whose families chose to donate were more likely to have had significant clinical symptoms and more severe pathology. Although ascertainment could bias the estimated effects, we have previously shown that selection bias does not negate associations in the UNITE brain bank, at least with respect to RHI-CTE relationships [2, 77]. The current sample consisted of predominantly white men who played football and inferences to women and other populations exposed to RHI cannot be made. The study also lacked disease comparison groups that are needed in future research to better understand the specificity of our results.

## Conclusions

In conclusion, global and cortical p-tau density were associated with informant-reported cognitive and functional symptoms in an autopsy sample of brain donors with CTE. Regional p-tau aggregates, particularly in the FC, were associated with symptoms of neurobehavioral dysregulation. These findings can inform future iterations of the TES criteria, which are intended to reflect the clinical syndrome of underlying CTE pathology. This study supports continued inclusion of cognitive impairment as a core feature of the TES criteria. While there was support for neurobehavioral dysregulation, effects were weaker and inconsistent across scales. TES criteria were developed without in vivo biomarkers and integration of biomarkers, particularly that target the FC, will be important for improving TES specificity in future iterations.

### Supplementary Information

**Additional file 1: Supplemental Table 1.** Sample characteristics of brain donors with CTE-only. **Supplemental Table 2.** Semi-quantitative ratings of p-tau severity in brain donors with CTE-only. **Supplemental Table 3.** Descriptive statistics of informant-completed standardized clinical scales. **Supplemental Table 4.** Association between regional p-tau and cognitive and daily function scales: summary of multivariable OLS regression adjusted for CERAD neuritic amyloid plaque score, limbic/neocortical LBD and FTLD. **Supplemental Table 5.** Association between regional p-tau and scales of neurobehavioral dysregulation, depression, and apathy: summary of multivariable OLS regression results adjusted for CERAD neuritic amyloid plaque score, limbic/neocortical LBD and FTLD. **Supplemental Table 6.** Association between regional p-tau and standardized scales: summary of ridge regression coefficients adjusted for CERAD neuritic amyloid plaque score, limbic/neocortical LBD and FTLD. **Supplemental Table 7.** Association between regional p-tau and cognitive and daily function scales: summary of multivariable OLS regression results in brain donors with CTE-only. **Supplemental Table 8.** Association between regional p-tau and scales of neurobehavioral dysregulation, depression, and apathy: summary of multivariable OLS regression results in brain donors with CTE-only. **Supplemental Table 9.** Association between regional p-tau and standardized scales: summary of ridge regression coefficients in brain donors with CTE-only. **Supplemental Figure 1.** Pairwise Correlation Matrix of the Regional P-tau Semi-Quantitative Rating Scales and Age at Death. **Supplemental Figure 2.** Factor Analysis of the Cognitive Difficulties Scales.

## Data Availability

The data generated and analyzed for the current study are available in the FITBIR repository: https://fitbir.nih.gov/study_profile/438. Requests may also be made to the corresponding author.

## Abbreviations

Aß: Beta amyloid
AD: Alzheimer’s disease
AD/ADRD: Alzheimer’s disease and related dementias
AES: Apathy Evaluation Scale
AIC: Akaike information criterion
ALS: Amyotrophic lateral sclerosis
AT8: P-tau stain
BIS-11: Barratt Impulsiveness Scale
BRIEF-A: Behavior Rating Inventory of Executive Function-Adult Version
BU: Boston University
BUMC: Boston University Medical Campus
bvFTD: Behavioral variant frontotemporal dementia
CDS: Cognitive Difficulties Scale
CERAD: Consortium to Establish a Registry for AD
CFI: Comparative fit index
CI: Confidence interval
CLF: Concussion Legacy Foundation
CTE: Chronic traumatic encephalopathy
DLFC: Dorsolateral frontal cortex
EC: Entorhinal cortex
FAQ: Functional Activities Questionnaire
FC: Frontal cortex
FDR: False discovery rate
FTD: Frontotemporal dementia
FTLD: Frontotemporal lobar degeneration
GDS: Geriatric Depression Scale
GPCM: Generalized partial credit model
HC: Hippocampal composite
IFC: Inferior frontal cortex
IPC: Inferior parietal cortex
IRB: Institutional review board
LBD: Lewy body disease
LC: Locus coeruleus
LHE: Luxol fast blue, hematoxylin and eosin
MCI: Mild cognitive impairment
MI: Metacognition Index
MIRT: Multidimensional item response theory
MND: Motor neuron disease
MTL: Medial temporal lobe
NINDS-NIBIB: National Institute on Neurological Disorders and Stroke—National Institute of Biomedical Imaging and Bioengineering (NIBIB)
NFT: Neurofibrillary tangles
NRM: Nominal response model
OLS: Ordinary least squares
OSA: Obstructive sleep apnea
pTDP-43: Phosphorylated TDP-43
PET: Positron emission tomography
P-tau: Hyperphosphorylated tau
RHI: Repetitive head impact
RMSEA: Root mean square error of approximation
STC: Superior temporal cortex
TBI: Traumatic brain injury
TES: Traumatic encephalopathy syndrome
TLI: Tucker Lewis Index
UNITE: Understanding Neurological Injury and Traumatic Encephalopathy
VA: Veteran’s Affairs
