# Supplementary material for: Cognitive, functional, and neuropsychiatric correlates of regional tau pathology in autopsy-confirmed chronic traumatic encephalopathy
Source: Mol Neurodegener. 2024 Feb 6;19:10. doi: 10.1186/s13024-023-00697-2 (PMC10845638; doi:10.1186/s13024-023-00697-2)
Supplement: Supplementary file 1 — Additional file 1: Supplemental Table 1. Sample characteristics of brain donors with CTE-only. Supplemental Table 2. Semi-quantitative ratings of p-tau severity in brain donors with CTE-only. Supplemental Table 3. Descriptive statistics of informant-completed standardized clinical scales. Supplemental Table 4. Association between regional p-tau and cognitive and daily function scales: summary of multivariable OLS regression adjusted for CERAD neuritic amyloid plaque score, limbic/neocortical LBD and FTLD. Supplemental Table 5. Association between regional p-tau and scales of neurobehavioral dysregulation, depression, and apathy: summary of multivariable OLS regression results adjusted for CERAD neuritic amyloid plaque score, limbic/neocortical LBD and FTLD. Supplemental Table 6. Association between regional p-tau and standardized scales: summary of ridge regression coefficients adjusted for CERAD neuritic amyloid plaque score, limbic/neocortical LBD and FTLD. Supplemental Table 7. Association between regional p-tau and cognitive and daily function scales: summary of multivariable OLS regression results in brain donors with CTE-only. Supplemental Table 8. Association between regional p-tau and scales of neurobehavioral dysregulation, depression, and apathy: summary of multivariable OLS regression results in brain donors with CTE-only. Supplemental Table 9. Association between regional p-tau and standardized scales: summary of ridge regression coefficients in brain donors with CTE-only. Supplemental Figure 1. Pairwise Correlation Matrix of the Regional P-tau Semi-Quantitative Rating Scales and Age at Death. Supplemental Figure 2. Factor Analysis of the Cognitive Difficulties Scales. [file 13024_2023_697_MOESM1_ESM.pdf]

Supplemental Table 1. Sample characteristics of brain donors with CTE-only

	**CTE-Only (n=234)**
**Demographics**	
Age of death, mean (SD) years	56.33 (19.61)
Race, n (%) Black	44 (18.8)
Education Level, n (%)	
Some High School	1 (0.4)
High School Diploma/GED	3 (1.3)
Some College	60 (25.6)
College Degree	121 (51.7)
More than College	9 (3.8)
Graduate Degree	40 (17.1)
**Athletics**	
Sport Played, n (%)[a]	
Football	218 (93.2)
Ice hockey	21 (9.0)
Wrestling	23 (9.8)
Soccer	22 (9.4)
Boxing	11 (4.7)
Skiing	0
Rugby	8 (3.4)
Lacrosse	8 (3.4)
Other	4 (1.7)
Years of Football Play, mean (SD)	13.96 (5.50)
Highest Level Football Played, n (%)	
Youth	5 (2.3)
High school	19 (8.7)
College	65 (29.8)
Semi-Professional	6 (2.8)
Professional	123 (56.4)
Military history, n (%)	44 (18.8)
**Medical Characteristics, n (%)**	
Hypertension	121 (52.6)
Obstructive sleep apnea	59 (25.9)
Substance use treatment	66 (28.4)
**Neuropathological Characteristics**	
CTE stage, n (%)	
Stage I	61 (26.1)
Stage II	52 (22.2)
Stage III	91 (38.9)
Stage IV	30 (12.8)

Note. Sample included brain donors who had CTE and no other neurodegenerative disease, defined by Alzheimer's disease, Lewy body disease, frontotemporal lobar degeneration, and/or motor neuron disease. [a]Categories are not mutually exclusive
Abbreviations: CTE = chronic traumatic encephalopathy

Supplemental Table 2. Semi-quantitative ratings of p-tau severity in brain donors with CTE-only

	**CTE-only**<br>**N = 234**
**Dorsolateral frontal cortex, n=231**	
None	26 (11.3)
Mild	80 (34.6)
Moderate	68 (29.4)
Severe	57 (24.7)
**Inferior frontal cortex, n=225**	
None	70 (31.1)
Mild	92 (40.9)
Moderate	42 (18.7)
Severe	21 (9.3)
**Superior temporal cortex, n=230**	
None	52 (22.6)
Mild	68 (29.6)
Moderate	66 (28.7)
Severe	44 (19.1)
**Inferior parietal cortex, n=229**	
None	75 (32.8)
Mild	87 (38.0)
Moderate	39 (17.0)
Severe	28 (12.2)
**CA1-hippocampus, n=228**	
None	58 (25.4)
Mild	84 (36.8)
Moderate	38 (16.7)
Severe	48 (21.1)
**CA2-hippocampus, n=225**	
None	85 (37.8)
Mild	59 (26.2)
Moderate	45 (20.0)
Severe	36 (16.0)
**CA4-hippocampus, n=228**	
None	84 (36.8)
Mild	86 (37.7)
Moderate	26 (11.4)
Severe	32 (14.0)
**Entorhinal cortex, n=231**	
None	36 (15.6)
Mild	53 (22.9)
Moderate	73 (31.6)
Severe	69 (29.9)
**Amygdala, n=229**	
None	45 (19.7)
Mild	85 (37.1)
Moderate	58 (25.3)
Severe	41 (17.9)
**Locus coeruleus, n=217**	
None	19 (8.8)
Mild	42 (19.4)
Moderate	88 (40.6)
Severe	68 (31.3)

Note. Sample included brain donors who had CTE and no other neurodegenerative disease, defined by Alzheimer's disease, Lewy body disease, frontotemporal lobar degeneration, and/or motor neuron disease. Sample sizes vary due to missing data. Abbreviations: CTE = chronic traumatic encephalopathy

Supplemental Table 3. Descriptive statistics of informant-completed standardized clinical scales

	CTE-only		
	**Mean**	**SD**	**Impaired n (%)**
**Cognitive Function**			
CDS, n=215	78.65	42.37	--
BRIEF-A MI, n=215	85.50	20.99	153 (71.2)
**Daily Function**			
FAQ, n=224	10.77	10.55	100 (44.6)
**Neurobehavioral Dysregulation**			
BIS-11, n=213	74.01	16.1	--
BRIEF-A BRI, n=215	65.27	15.23	156 (72.6)
Brown-Goodwin-Adult Sum, n=203	18.16	6.30	--
**Depression and Apathy**			
GDS-15, n=217	8.94	4.49	169 (77.9)
AES, n=211	46.34	13.96	164 (77.7)

Note. Sample included brain donors who had CTE and no other neurodegenerative disease, defined by Alzheimer's disease, Lewy body disease, frontotemporal lobar degeneration, and/or motor neuron disease. Sample sizes vary across scales due to missing data. BRIEF-A MI and BRI are T-scores and a T≥65 reflects clinically meaningful symptoms of executive dysfunction and behavioral dysregulation, respectively. A score of 9 or higher on the FAQ is indicative of functional impairment and scores of 5 and 34 or higher on the GDS-15 and AES represent clinically meaningful symptoms of depression and apathy, respectively. For the remaining scales, strongly supported cutoffs have not been identified in the literature.

Abbreviations: CTE = chronic traumatic encephalopathy, CDS = Cognitive Difficulties Scale, BRIEF-A = Behavior Rating Inventory of Executive Function for Adults, MI = Metacognition Index, FAQ = Functional Activities Questionnaire, BIS-11 = Barratt Impulsiveness Scale, GDS = Geriatric Depression Scale, 15-item version, AES = Apathy Evaluation Scale

Supplemental Table 4. Association between regional p-tau and cognitive and daily function scales: summary of multivariable OLS regression adjusted for CERAD neuritic amyloid plaque score, limbic/neocortical LBD and FTLD.

	CDS Total Score (n = 282)			CDS Attention Factor Score (n = 282)			CDS Memory Factor Score (n = 282)			CDS Language Factor Score (n = 282)			CDS Motor Factor Score (n = 282)			BRIEF-A MI (n = 283)			FAQ (n = 288)		
**P-tau ratings**	**b**	**95% CI**	**p**	**b**	**95% CI**	**p**	**beta**	**95% CI**	**p**	**b**	**95% CI**	**p**	**b**	**95% CI**	**p**	**b**	**95% CI**	**p**	**b**	**95% CI**	**p**
Frontal	0.18	0.06, 0.29	<0.01	0.20	0.08, 0.32	<0.01	0.16	0.04, 0.28	0.03	0.16	0.04, 0.28	0.04	0.13	0.01, 0.25	0.09	0.19	0.06, 0.33	0.05	0.15	0.05, 0.26	<0.01
Superior temporal	0.08	-0.03, 0.19	0.40	0.10	-0.02, 0.21	0.22	0.08	-0.03, 0.20	0.43	0.08	-0.03, 0.2	0.31	0.04	-0.08, 0.15	0.67	0.03	-0.1, 0.16	0.86	0.12	0.02, 0.22	0.03
Inferior parietal	0.15	0.03, 0.26	0.03	0.18	0.06, 0.3	<0.01	0.16	0.04, 0.27	0.03	0.13	0.01, 0.25	0.10	0.15	0.03, 0.27	0.04	0.11	-0.03, 0.24	0.34	0.12	0.02, 0.22	0.05
Amygdala	0.07	-0.06, 0.19	0.56	0.11	-0.02, 0.24	0.22	0.03	-0.09, 0.16	0.82	0.09	-0.04, 0.21	0.34	0.11	-0.02, 0.24	0.18	0.08	-0.06, 0.23	0.58	0.13	0.02, 0.24	0.05
Entorhinal	-0.01	-0.13, 0.11	0.93	0.01	-0.11, 0.13	0.92	-0.06	-0.17, 0.07	0.80	0.00	-0.12, 0.12	0.99	0.06	-0.06, 0.18	0.60	-0.01	-0.15, 0.13	0.9	0.07	-0.04, 0.17	0.40
Hippocampus	-0.03	-0.16, 0.09	0.75	-0.02	-0.14, 0.11	0.92	-0.07	-0.2, 0.05	0.53	-0.05	-0.18, 0.07	0.65	0.07	-0.06, 0.19	0.51	0.01	-0.13, 0.15	0.84	0.03	-0.07, 0.14	0.80
Locus coereulus	0.00	-0.11, 0.11	0.98	-0.01	-0.12, 0.1	0.92	-0.02	-0.13, 0.09	0.82	-0.02	-0.13, 0.09	0.80	0.04	-0.07, 0.15	0.67	0.02	-0.1, 0.15	0.86	0.03	-0.06, 0.12	0.80

Note. Estimates are standardized betas. Regional p-tau severity was rated on a 0-3 scale with 0 being none and 3 being severe. Frontal is a summary composite of the dorsolateral frontal cortex and the inferior frontal cortex. CA1, CA2, and CA4 were summed to create the hippocampus composite. For all clinical scales, higher scores are worse. P-values were false discovery rate adjusted using the Benjamini-Hochberg procedure. Models were adjusted for age at death, racial identity, education level, history of hypertension and obstructive sleep apnea, history of substance use treatment, CERAD neuritic amyloid plaque score, limbic/neocortical LBD and FTLD.

Abbreviations: CERAD = Consortium to Establish a Registry for Alzheimer's disease, LBD = Lewy Body Disease, FTLD = frontemporal lobar degeneration, OLS = ordinary least squares, CDS = Cognitive Difficulties Scale, BRIEF-A = Behavior Rating Inventory of Executive Function for Adults, MI = Metacognition Index, FAQ = Functional Activities Questionnaire

Supplemental Table 5. Association between regional p-tau and scales of neurobehavioral dysregulation, depression, and apathy: summary of multivariable OLS regression results adjusted for CERAD neuritic amyloid plaque score, limbic/neocortical LBD and FTLD.

	BRIEF-A BRI (n = 283)			BIS-11 (n = 281)			Brown Goodwin (n = 261)			GDS-15 (n = 284)			AES (n = 279)		
**P-tau ratings**	**b**	**95% CI**	**p**	**b**	**95% CI**	**p**	**b**	**95% CI**	**p**	**b**	**95% CI**	**p**	**b**	**95% CI**	**p**
Frontal	0.16	0.02, 0.3	0.13	0.12	-0.02, 0.26	0.6	-0.06	-0.2, 0.08	0.56	0.06	-0.08, 0.20	0.71	0.06	-0.08, 0.2	0.81
Superior temporal	0.05	-0.08, 0.19	0.8	0.03	-0.1, 0.16	0.97	-0.06	-0.19, 0.08	0.56	0.02	-0.11, 0.15	0.82	0.03	-0.1, 0.16	0.81
Inferior parietal	0.05	-0.09, 0.19	0.8	0.02	-0.12, 0.16	0.97	-0.06	-0.2, 0.08	0.56	0.04	-0.09, 0.18	0.82	0.07	-0.07, 0.20	0.81
Amygdala	0.01	-0.14, 0.16	0.91	0.05	-0.1, 0.19	0.97	0.06	-0.09, 0.21	0.57	0.02	-0.13, 0.17	0.82	0.08	-0.06, 0.23	0.81
Entorhinal	-0.04	-0.18, 0.1	0.8	0.03	-0.11, 0.16	0.97	0.02	-0.12, 0.16	0.75	0.07	-0.06, 0.21	0.58	-0.01	-0.14, 0.13	0.92
Hippocampus	0.04	-0.1, 0.19	0.8	0.00	-0.14, 0.14	0.97	0.10	-0.04, 0.24	0.38	0.11	-0.03, 0.25	0.43	0.01	-0.13, 0.15	0.92
Locus coereulus	0.02	-0.11, 0.15	0.8	-0.01	-0.13, 0.12	0.97	0.04	-0.09, 0.17	0.57	0.07	-0.1, 0.15	0.82	0.02	-0.1, 0.15	0.83

Note. Estimates are standardized betas. Regional p-tau severity was rated on a 0-3 scale with 0 being none and 3 being severe. Frontal is a summary composite of the dorsolateral frontal cortex and the inferior frontal cortex. CA1, CA2, and CA4 were summed to create the hippocampus composite. For all clinical scales, higher scores are worse. P-values were false discovery rate adjusted using the Benjamini-Hochberg procedure. Models were adjusted for age at death, racial identity, education level, history of hypertension and obstructive sleep apnea, history of substance use treatment, CERAD neuritic amyloid plaque score, limbic/neocortical LBD and FTLD.

Abbreviations: CERAD = Consortium to Establish a Registry for Alzheimer's disease, LBD = Lewy Body Disease, FTLD = frontemporal lobar degeneration, OLS = ordinary least squares, BRIEF-A = Behavior Rating Inventory of Executive Function for Adults, BRI = Behavioral Regulation Index, BIS-11 = Barratt Impulsiveness Scale, GDS = Geriatric Depression Scale, 15-item version, AES = Apathy Evaluation Scale

Supplemental Table 6. Association between regional p-tau and standardized scales: summary of ridge regression coefficients adjusted for CERAD neuritic amyloid plaque score, limbic/neocortical LBD and FTLD.

	CDS Total		CDS-Attention		CDS. Memory		CDS Language		CDS Motor		BRIEF-A MI		FAQ		BRIEF-A BRI		BIS-11		Brown-Goodwin		GDS-15		AES	
	**Ridge coef**	**95% CI**	**Ridge coef**	**95% CI**	**Ridge coef**	**95% CI**	**Ridge coef**	**95% CI**	**Ridge coef**	**95% CI**	**Ridge coef**	**95% CI**	**Ridge coef**	**95% CI**	**Ridge coef**	**95% CI**	**Ridge coef**	**95% CI**	**Ridge coef**	**95% CI**	**Ridge coef**	**95% CI**	**Ridge coef**	**95% CI**
Frontal	0.14	0.06, 0.22	0.12	0.06, 0.18	0.14	0.04, 0.24	0.12	0.05, 0.2	0.07	0.02, 0.12	0.12	0.04, 0.19	0.09	0.02, 0.16	0.06	0.01, 0.1	0.04	0.01, 0.09	-0.03	-0.08, 0.03	0.00	-0.02, 0.01	0.03	-0.01, 0.07
Superior temporal	0.01	-0.07, 0.10	0.02	-0.05, 0.09	0.03	-0.05, 0.12	0.03	-0.04, 0.12	-0.01	-0.07, 0.05	-0.01	-0.08, 0.05	0.05	-0.02, 0.13	0.01	-0.03, 0.05	0.00	-0.04, 0.06	-0.04	-0.10, 0.02	0.00	-0.02, 0.02	0.02	-0.01, 0.06
Inferior parietal	0.09	0.01, 0.18	0.09	0.01, 0.15	0.11	0.02, 0.21	0.07	0.00, 0.15	0.08	0.02, 0.14	0.05	-0.01, 0.12	0.05	-0.03, 0.14	0.01	-0.03, 0.06	-0.01	-0.05, 0.03	-0.04	-0.10, 0.02	0.00	-0.02, 0.02	0.03	-0.01, 0.7
Amygdala	0.05	-0.04, 0.14	0.07	0.01, 0.15	0.04	-0.06, 0.14	0.08	0.00, 0.17	0.06	0.00, 0.12	0.04	-0.03, 0.11	0.09	0.02, 0.16	-0.01	-0.05, 0.04	0.00	-0.04, 0.04	0.01	-0.06, 0.06	-0.01	-0.03, 0.01	0.04	0.01, 0.07
Entorhinal	-0.05	-0.14, 0.04	-0.03	-0.11, 0.04	-0.07	-0.16, 0.03	-0.03	-0.11, 0.04	0.01	-0.05, 0.07	-0.04	-0.12, 0.02	0.00	-0.08, 0.07	-0.03	-0.07, 0.01	0.00	-0.04, 0.04	0.00	-0.05, 0.06	0.00	-0.02, 0.02	0.00	-0.03, 0.04
Hippocampus	-0.03	-0.11, 0.06	-0.02	-0.09, 0.05	-0.05	-0.14, 0.06	-0.05	-0.13, 0.03	0.03	-0.04, 0.09	0.00	-0.06, 0.07	-0.01	-0.09, 0.06	0.00	-0.05, 0.04	-0.02	-0.06, 0.02	0.04	-0.01, 0.11	0.00	-0.02, 0.02	0.01	-0.03, 0.04
Locus coeruleus	-0.03	-0.1, 0.04	-0.04	-0.11, 0.02	-0.03	-0.1, 0.05	-0.04	-0.11, -0.03	-0.01	-0.07, 0.04	-0.01	-0.07, 0.06	-0.02	-0.1, 0.04	0.01	-0.03, 0.06	0.00	-0.04, 0.05	0.01	-0.06, 0.09	0.00	-0.03, 0.02	0.01	-0.03, 0.05

Note. To investigate which regions may be driving the associations, independent of the effects from the other regions, multivariable ridge regression models were performed for the regional scales of p-tau severity. Ridge regression is used when there are many independent variables that are highly correlated. A separate ridge regression was performed for each clinical scale. Estimates are standardized betas. Models were adjusted for age at death, racial identity, education level, history of hypertension, obstructive sleep apnea, history of substance use treatment, CERAD neuritic amyloid plaque score, limbic/neocortical LBD and FTLD. Frontal is a summary composite of the dorsolateral frontal cortex and the inferior frontal cortex. CA1, CA2, and CA4 were summed to create the hippocampus composite. For all clinical scales, higher scores are worse.

Abbreviations: CERAD = Consortium to Establish a Registry for Alzheimer's disease, LBD = Lewy Body Disease, FTLD = frontemporal lobar degeneration, CDS = Cognitive Difficulties Scale, BRIEF-A = Behavior Rating Inventory of Executive Function for Adults, MI = Metacognition Index, FAQ = Functional Activities Questionnaire, BRI = Behavioral Regulation Index, BIS-11 = Barratt Impulsiveness Scale, GDS = Geriatric Depression Scale, 15-item version, AES = Apathy Evaluation Scale

Supplemental Table 7. Association between regional p-tau and cognitive and daily function scales: summary of multivariable OLS regression results in brain donors with CTE-only

	CDS Total Score (n = 184)			CDS Attention Factor Score (n = 184)			CDS Memory Factor Score (n = 184)			CDS Language Factor Score (n = 184)			CDS Motor Factor Score (n = 184)			BRIEF-A MI (n = 185)			FAQ (n = 188)		
**P-tau ratings**	**b**	**95% CI**	**p**	**b**	**95% CI**	**p**	**b**	**95% CI**	**p**	**b**	**95% CI**	**p**	**b**	**95% CI**	**p**	**b**	**95% CI**	**p**	**b**	**95% CI**	**p**
Frontal	0.17	0.03, 0.31	0.14	0.18	0.03, 0.32	0.14	0.15	0.01, 0.29	0.28	0.16	0.02, 0.31	0.14	0.07	-0.07, 0.22	0.64	0.20	0.04, 0.35	0.14	0.15	0.02, 0.27	0.14
Superior temporal	0.05	-0.09, 0.2	0.79	0.06	-0.09, 0.2	0.65	0.07	-0.08, 0.21	0.83	0.04	-0.11, 0.18	0.81	-0.01	-0.15, 0.14	0.97	0.02	-0.14, 0.17	0.89	0.09	-0.04, 0.21	0.40
Inferior parietal	0.08	-0.06, 0.22	0.76	0.10	-0.05, 0.24	0.65	0.09	-0.05, 0.23	0.59	0.10	-0.04, 0.24	0.48	0.00	-0.14, 0.14	0.97	0.07	-0.08, 0.22	0.70	0.06	-0.06, 0.19	0.58
Amygdala	0.07	-0.1, 0.24	0.79	0.09	-0.08, 0.26	0.65	0.05	-0.12, 0.22	0.83	0.13	-0.04, 0.3	0.46	0.11	-0.06, 0.28	0.61	0.14	-0.04, 0.32	0.61	0.14	0.00, 0.28	0.23
Entorhinal	-0.07	-0.23, 0.1	0.79	-0.05	-0.22, 0.12	0.65	-0.11	-0.27, 0.06	0.59	-0.03	-0.2, 0.13	0.81	-0.04	-0.21, 0.12	0.97	- 0.03	-0.21, 0.15	0.89	-0.04	-0.18, 0.1	0.73
Hippocampus	0.04	-0.13, 0.22	0.79	0.06	-0.12, 0.24	0.65	0.02	-0.16, 0.19	0.93	0.08	-0.1, 0.25	0.78	0.10	-0.07, 0.28	0.61	0.07	-0.12, 0.27	0.70	-0.02	-0.16, 0.13	0.84
Locus coereulus	0.01	-0.14, 0.17	0.92	0.00	-0.15, 0.16	0.96	0.01	-0.15, 0.16	0.93	0.03	-0.13, 0.18	0.81	-0.01	-0.17, 0.14	0.97	0.10	-0.07, 0.27	0.67	-0.05	-0.19, 0.08	0.63

Note. Sample included brain donors who had CTE and no other neurodegenerative disease, defined by Alzheimer's disease, Lewy body disease, frontotemporal lobar degeneration, and/or motor neuron disease. Estimates are standardized betas. Regional p-tau severity was rated on a 0-3 scale with 0 being none and 3 being severe. Frontal is a summary composite of the dorsolateral frontal cortex and the inferior frontal cortex. CA1, CA2, and CA4 were summed to create the hippocampus composite. For all clinical scales, higher scores are worse. P-values were false discovery rate adjusted using the Benjamini-Hochberg procedure. Models were adjusted for age at death, racial identity, education level, history of hypertension and obstructive sleep apnea, and history of substance use treatment.

Abbreviations: OLS = ordinary least squares, BRIEF-A = Behavior Rating Inventory of Executive Function for Adults, BRI = Behavioral Regulation Index, BIS-11 = Barratt Impulsiveness Scale, GDS = Geriatric Depression Scale, 15-item version, AES = Apathy Evaluation Scale

Supplemental Table 8. Association between regional p-tau and scales of neurobehavioral dysregulation, depression, and apathy: summary of multivariable OLS regression results in brain donors with CTE-only

	**BRIEF-A BRI** (n = 185)			**BIS-11** (n = 183)			**Brown Goodwin** (n = 170)			**GDS-15** (n = 186)			**AES** (n = 181)		
**P-tau ratings**	**b**	**95% CI**	**p**	**b**	**95% CI**	**p**	**b**	**95% CI**	**p**	**b**	**95% CI**	**p**	**b**	**95% CI**	**p**
Frontal	0.18	0.03, 0.33	0.14	0.09	-0.06, 0.24	0.69	-0.08	-0.24, 0.07	0.56	0.05	-0.1, 0.2	0.77	0.09	-0.06, 0.24	0.73
Superior temporal	0.03	-0.12, 0.18	0.90	0.00	-0.15, 0.15	0.98	-0.06	-0.22, 0.09	0.62	0.00	-0.15, 0.15	0.98	0.03	-0.12, 0.18	0.93
Inferior parietal	0.05	-0.10, 0.20	0.79	-0.05	-0.19, 0.1	0.69	-0.06	-0.21, 0.09	0.62	-0.03	-0.17, 0.12	0.90	0.02	-0.13, 0.16	0.93
Amygdala	0.04	-0.14, 0.23	0.88	0.07	-0.11, 0.25	0.69	0.06	-0.12, 0.25	0.62	0.01	-0.17, 0.18	0.98	0.12	-0.06, 0.3	0.63
Entorhinal	0.01	-0.16, 0.19	0.91	0.08	-0.1, 0.25	0.69	0.08	-0.1, 0.26	0.62	0.06	-0.11, 0.23	0.77	-0.02	-0.19, 0.16	0.93
Hippocampus	0.15	-0.04, 0.34	0.39	0.12	-0.06, 0.3	0.69	0.12	-0.06, 0.31	0.44	0.14	-0.04, 0.32	0.36	0.03	-0.15, 0.22	0.93
Locus coereulus	0.08	-0.09, 0.25	0.68	0.06	-0.1, 0.22	0.69	0.03	-0.13, 0.2	0.70	0.05	-0.11, 0.21	0.77	0.00	-0.16, 0.17	0.96

Note. Sample included brain donors who had CTE and no other neurodegenerative disease, defined by Alzheimer's disease, Lewy body disease, frontotemporal lobar degeneration, and/or motor neuron disease. Estimates are standardized betas. Regional p-tau severity was rated on a 0-3 scale with 0 being none and 3 being severe. Frontal is a summary composite of the dorsolateral frontal cortex and the inferior frontal cortex. CA1, CA2, and CA4 were summed to create the hippocampus composite. For all clinical scales, higher scores are worse. P-values were false discovery rate adjusted using the Benjamini-Hochberg procedure. Models were adjusted for age at death, racial identity, education level, history of hypertension and obstructive sleep apnea, and history of substance use treatment.

Abbreviations: OLS = ordinary least squares, BRIEF-A = Behavior Rating Inventory of Executive Function for Adults, BRI = Behavioral Regulation Index, BIS-11 = Barratt Impulsiveness Scale, GDS = Geriatric Depression Scale, 15-item version, AES = Apathy Evaluation Scale

Supplemental Table 9. Association between regional p-tau and standardized scales: summary of ridge regression coefficients in brain donors with CTE-only

	CDS Total		CDS-Attention		CDS. Memory		CDS Language		CDS Motor		BRIEF-A MI		FAQ		BRIEF-A BRI		BIS-11		Brown-Goodwin		GDS-15		AES	
	**Ridge coef**	**95% CI**	**Ridge coef**	**95% CI**	**Ridge coef**	**95% CI**	**Ridge coef**	**95% CI**	**Ridge coef**	**95% CI**	**Ridge coef**	**95% CI**	**Ridge coef**	**95% CI**	**Ridge coef**	**95% CI**	**Ridge coef**	**95% CI**	**Ridge coef**	**95% CI**	**Ridge coef**	**95% CI**	**Ridge coef**	**95% CI**
Frontal	0.09	0.02, 0.15	0.10	0.04, 0.16	0.10	0.00, 0.19	0.08	0.02, 0.15	0.05	-0.02, 0.12	0.02	0.01, 0.04	0.17	0.04, 0.31	0.05	0.01, 0.1	0.04	-0.02, 0.10	-0.07	-0.15, 0.02	0.02	-0.03, 0.07	0.04	-0.02, 0.11
Superior temporal	0.01	-0.06, 0.07	0.01	-0.05, 0.08	0.02	-0.06, 0.09	0.00	-0.06, 0.07	-0.01	-0.08, 0.07	0.00	-0.01, 0.01	0.03	-0.10, 0.18	-0.01	-0.04, 0.03	-0.02	-0.09, 0.05	-0.06	-0.17, 0.04	-0.01	-0.06, 0.05	0.01	-0.04, 0.06
Inferior parietal	0.01	-0.06, 0.07	0.02	-0.05, 0.09	0.03	-0.06, 0.12	0.02	-0.05, 0.08	-0.02	-0.08, 0.05	0.00	-0.02, 0.01	-0.03	-0.16, 0.10	0.00	-0.04, 0.05	-0.05	-0.11, 0.01	-0.03	-0.12, 0.05	-0.03	-0.09, 0.03	-0.01	-0.07, 0.05
Amygdala	0.05	-0.01, 0.11	0.05	-0.02, 0.11	0.05	-0.04, 0.15	0.07	0.01, 0.13	0.08	0.01, 0.16	0.01	-0.01, 0.02	0.18	0.02, 0.34	-0.02	-0.07, 0.02	0.00	-0.06, 0.07	0.01	-0.09, 0.10	-0.03	-0.08, 0.03	0.06	0.01, 0.12
Entorhinal	-0.04	-0.11, 0.01	-0.03	-0.09, 0.04	-0.09	-0.19, 0.01	-0.02	-0.07, 0.03	-0.03	-0.10, 0.04	0.00	-0.01, 0.02	-0.14	-0.28, -0.02	-0.02	-0.07, 0.02	0.00	-0.06, 0.06	0.04	-0.04, 0.14	0.00	-0.06, 0.06	-0.01	-0.08, 0.05
Hippocampus	0.05	-0.02, 0.12	0.05	-0.02, 0.12	0.05	-0.05, 0.16	0.06	0.00, 0.13	0.08	0.00, 0.17	0.01	0.00, 0.03	-0.04	-0.18, 0.10	0.01	-0.03, 0.04	0.01	-0.06, 0.07	0.04	-0.05, 0.13	0.02	-0.03, 0.08	0.02	-0.04, 0.08
Locus coeruleus	0.02	-0.05, 0.08	0.01	-0.07, 0.08	0.02	-0.06, 0.11	0.02	-0.04, 0.09	0.01	-0.06, 0.08	0.01	-0.01, 0.02	-0.08	-0.22, 0.05	0.02	-0.02, 0.07	0.02	-0.03, 0.08	0.02	-0.09, 0.13	0.02	-0.05, 0.08	0.02	-0.04, 0.08

Note. To investigate which regions may be driving the associations, independent of the effects from the other regions, multivariable ridge regression models were performed for the regional scales of p-tau severity. Ridge regression is used when there are many independent variables that are highly correlated. A separate ridge regression was performed for each clinical scale. Estimates are standardized betas. Models were adjusted for age at death, racial identity, education level, history of hypertension and obstructive sleep apnea, and history of substance use treatment. Frontal is a summary composite of the dorsolateral frontal cortex and the inferior frontal cortex. CA1, CA2, and CA4 were summed to create the hippocampus composite. For all clinical scales, higher scores are worse.

Abbreviations: CDS = Cognitive Difficulties Scale, BRIEF-A = Behavior Rating Inventory of Executive Function for Adults, MI = Metacognition Index, FAQ = Functional Activities Questionnaire, BRI = Behavioral Regulation Index, BIS-11 = Barratt Impulsiveness Scale, GDS = Geriatric Depression Scale, 15-item version, AES = Apathy Evaluation Scale

Supplemental Figure 1. Pairwise Correlation Matrix of the Regional P-tau Semi-Quantitative Rating Scales and Age at Death.

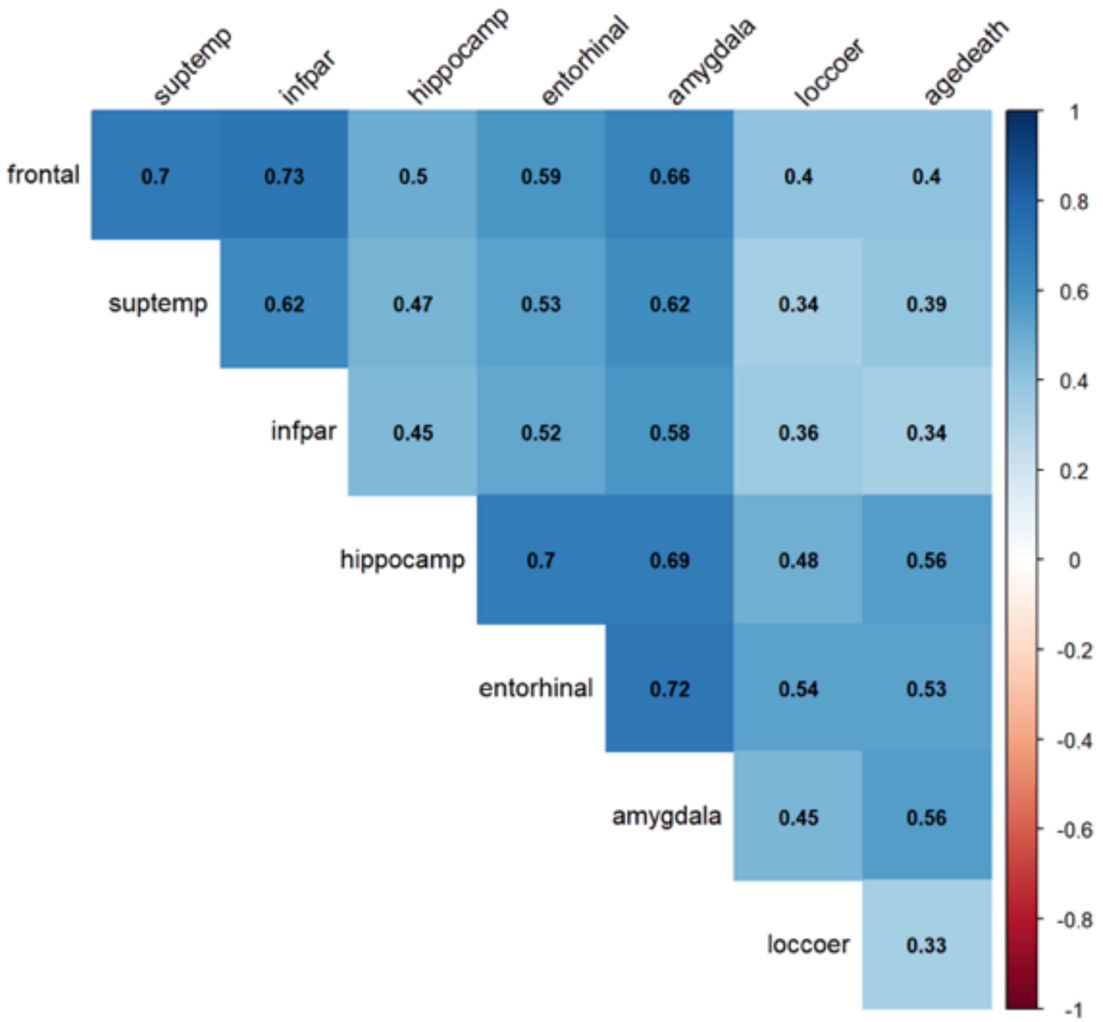

Supplemental Figure 2. Factor Analysis of the Cognitive Difficulties Scales.

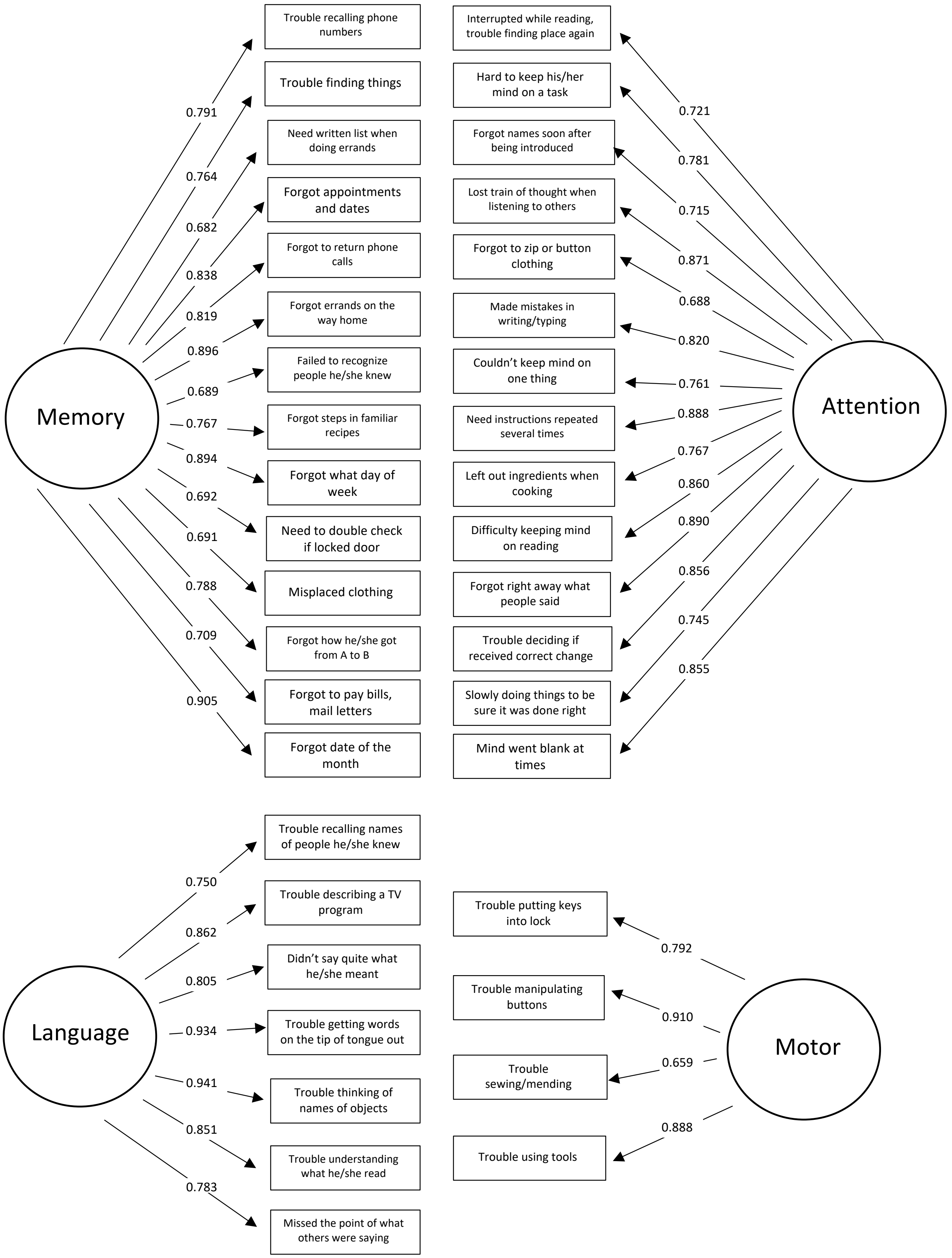

A confirmatory factor analysis of the cognitive difficulties scale (CDS) was performed to derive domain-level cognitive factor scores. A clinical neuropsychologist (MLA) and behavioral neurologist (JM) used their expert judgment to assign each item of the CDS to one of four cognitive domains, including attention, memory, language, and motor. A multidimensional item response theory (MIRT) model was used to derive the CDS factor scores. We used a 4-dimensional nominal response model as our final model and extracted the factor score estimates. The factors and item loadings are shown in the Figure.
